# A magnesium-induced RNA conformational switch at the internal ribosome entry site of hepatitis C virus genome visualized by atomic force microscopy

**DOI:** 10.1093/nar/gku1299

**Published:** 2014-12-15

**Authors:** Ana García-Sacristán, Miguel Moreno, Ascensión Ariza-Mateos, Elena López-Camacho, Rosa M. Jáudenes, Luis Vázquez, Jordi Gómez, José Ángel Martín-Gago, Carlos Briones

**Affiliations:** 1 Department of Molecular Evolution, Centro de Astrobiología (CSIC-INTA), Torrejón de Ardoz, Madrid 28850, Spain; 2 Centro de Investigaciones Biomédicas en Red de Enfermedades Hepáticas y Digestivas, (CIBERehd), Spain; 3 Laboratory of RNA Archaeology, Instituto de Parasitología y Biomedicina ‘López-Neyra’ (CSIC), Parque Tecnológico Ciencias de la Salud, Armilla, Granada 18016, Spain; 4 Instituto de Ciencia de Materiales de Madrid (CSIC), Cantoblanco, Madrid 28049, Spain

## Abstract

The 5′ untranslated region of hepatitis C virus (HCV) genomic RNA contains an internal ribosome entry site (IRES) element, composed of domains II–IV, which is required for cap-independent translation initiation. Little information on the 3D structure of the whole functional HCV IRES is still available. Here, we use atomic force microscopy to visualize the HCV IRES conformation in its natural sequence context, which includes the upstream domain I and the essential, downstream domains V and VI. The 574 nt-long molecule analyzed underwent an unexpected, Mg^2+^-induced switch between two alternative conformations: from ‘open’, elongated morphologies at 0–2 mM Mg^2+^ concentration to a ‘closed’, comma-shaped conformation at 4–6 mM Mg^2+^. This sharp transition, confirmed by gel-shift analysis and partial RNase T1 cleavage, was hindered by the microRNA miR-122. The comma-shaped IRES-574 molecules visualized at 4–6 mM Mg^2+^ in the absence of miR-122 showed two arms. Our data support that the first arm would contain domain III, while the second one would be composed of domains (I–II)+(V–VI) thanks to a long-range RNA interaction between the I-II spacer and the basal region of domain VI. This reinforces the previously described structural continuity between the HCV IRES and its flanking domains I, V and VI.

## INTRODUCTION

Hepatitis C virus (HCV) is the major etiological agent of chronic liver disease. There is no HCV vaccine and the traditional treatment based on a combination of alpha-interferon (IFN) and ribavirin (RBV) failed in about half of the patients. The need for new, alternative therapeutic approaches has encouraged the exploration of HCV life cycle as well as the development of direct-acting antiviral agents that have substantially increased sustained virologic response, what suggests that IFN-free regimens could lead to HCV eradication (1). With this aim, a thorough study of the native structure of the HCV genomic RNA is currently required, since certain structural/functional RNA elements, in particular those present at the 5′ and 3′ untranslated regions (UTR), are promising targets for antiviral therapy (2,3).

**Figure 1.**
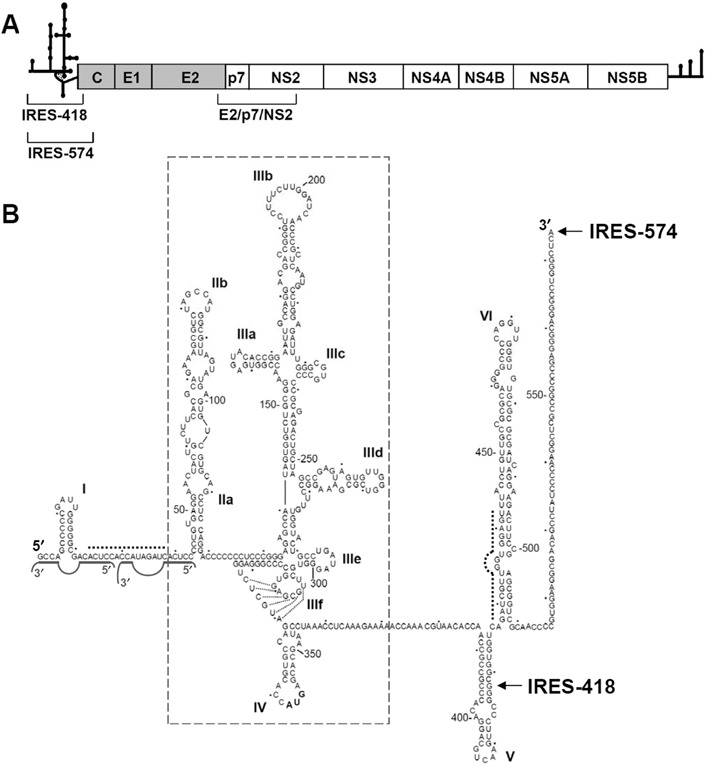
HCV IRES in its natural sequence context. (**A**) Schematic representation of the 9.6-Kb-long, ssRNA HCV genome. The secondary structures of the 5′ UTR and 3′ UTR are schematized. The structural (gray background) and non-structural (white background) proteins within the viral polyprotein are boxed. The regions corresponding to the IRES-418, IRES-574 and E2/p7/NS2 transcripts are shown. (**B**) Sequence and secondary structure of the 5′ UTR of HCV, previously deduced by different bioinformatic, biochemical and biophysical methods. Nucleotides are numbered every 50 residues, and domains/subdomains are labeled from I to VI. HCV IRES domains II to IV are boxed. The last residues of the IRES-418 and IRES-574 molecules used in this work are marked with arrows. Dashed lines show the complementary sequences at the I-II spacer (nts 24–38) and the basal region of domain VI (nts 428–442), while the two miR-122-binding sites are marked with solid gray lines.

The 5′ UTR of HCV is highly conserved among all the viral genotypes and contains an internal ribosome entry site (IRES) element that drives cap-independent initiation of translation of the viral polyprotein (4,5). The minimum sequence required for HCV IRES activity spans nucleotides (nts) 39–371 of the viral genome (6,7), and contains domains II to IV followed by the first 27 nts of the core coding sequence (4). Its secondary structure has been proposed using *in silico* RNA folding, covariation sequence analysis and biochemical methods (4,6,8–12) (Figure 1). The structures of individual HCV IRES domains or subdomains have been studied using techniques with atomic resolution (such as X-ray diffraction (XRD) and nuclear magnetic resonance (NMR)) (5,13–16), while electron microscopy (EM) has been used to visualize the structure of HCV IRES in its free form (17) and bound to the ribosome (18). A model of the HCV IRES structure in solution, based on small-angle X-ray scattering (SAXS) in combination with molecular dynamics simulations, has been also published (19).

HCV IRES is flanked upstream by a short stem-loop termed domain I, which is connected with domain II through a 23-nt-long ssRNA spacer region. Additionally, two downstream, structured domains termed V and VI (spanning nts 388–510 of the viral genome and located within the core coding sequence (20)), are essential for HCV viability (21) through a still unknown RNA structure-based mechanism. Phylogenetic (22), functional (23) and structural (24) studies have shown that a 15-nt-long sequence at the I-II spacer region can anneal with a complementary sequence at the basal region of domain VI (Figure 1), thus promoting a ‘closed’ conformation of the HCV IRES. This long-range RNA interaction is destabilized upon binding of the most abundant liver-specific microRNA, miR-122, which also interacts with the I-II spacer region by binding to two seed sites located 8 or 9 nts apart, depending on the viral isolate (25,26), and promotes the switch to an ‘open’ IRES conformation (27). However, the transition from ‘open’ to ‘closed’ (or vice versa) HCV IRES conformations has not been described in the absence of any effector RNA or protein molecule.

At the functional level, miR-122 binding has been associated to several effects including the increase of HCV RNA stability (28,29), reduction of the 5′ decay rates of the viral genome in infected cells (30) and stimulation of IRES-mediated translation (31). However, the distinct effects of miR-122 on translation observed *in vitro* and in cell culture (32,33) make it difficult to associate different HCV IRES conformations with miR-122 activities and to assess the role of miR-122 in the viral life cycle (34,35). This encourages the acquisition of additional structural and functional data on the interaction of miR-122 with HCV IRES in different experimental conditions.

RNA folding depends on the concentration of cations around the negatively charged phosphate backbone. Due to its high charge density, Mg^2+^ ions stabilize RNA tertiary structure more efficiently than other divalent or monovalent cations (36), thus being essential for the functional folding of large RNAs (37,38). The Mg^2+^ concentration required for optimal HCV IRES activity in translation-competent extracts ranges from 1 to 2.5 mM (39). HCV IRES can promote translation initiation at 2.5 mM Mg^2+^ without requiring the complete set of initiation factors, and it is able to drive initiation factor-independent translation at 5 mM Mg^2+^ (40). Although the Mg^2+^-dependent secondary structure of the minimal HCV IRES (domains II–IV) has already been investigated (10), new technological approaches are needed to visualize the effect of Mg^2+^ on the tertiary fold of the HCV IRES in its natural sequence context.

Atomic force microscopy (AFM) is a powerful nanotechnology-based tool for the structural analysis of a wide range of biological entities. It provides a 3D surface profile of the imaged sample without requiring any staining or coating, thus being less destructive and disruptive than EM. The nanometer resolution of this technique is optimal for the visualization of nucleic acid molecules (41–45), including RNA and RNA-protein complexes (46–49). AFM is increasingly being used in virology (50–53) and low-resolution images of the whole HCV genomic RNA have been published (54,55). However, AFM has not yet been applied to the structural characterization of either viral 5′ UTR regions or viral/cellular IRES elements. Here, we analyze by AFM the Mg^2+^-induced RNA folding of the HCV IRES in two constructions that contain either domains I-IV or I-VI. AFM results, in combination with gel-shift analysis and partial RNase T1 digestion, have allowed us to unveil a sharp magnesium-induced switch from an ‘open’ to a ‘closed’ conformation of the HCV IRES in its natural sequence context (domains I-VI), this transition being partially hindered by the presence of miR-122 in the medium.

## MATERIALS AND METHODS

### RNA synthesis

HCV RNA transcripts were obtained from the plasmid pN(1–4728) that contains the subgenomic region of HCV genotype 1b (GenBank accession number AF139594) spanning nts 1–4728, preceded by the T7 promoter (56). The IRES-418 construction (nts 1–418, IRES domains I-IV) and the E2/p7/NS2 fragment (nts 2478–3107) were obtained by polymerase chain reaction (PCR) amplification of pN(1–4728). The forward primer 1b/2a-5′-T71(+) (5′-TAATACGACTCACTATAGGACCCGCCCCGATTGGGGGCGA-3′, where the underlined sequence corresponds to the T7 RNA polymerase promoter), and the reverse primer 1b-C393(-) (5′-CCGGAATTCCCCGGGAACTTGACGTCCTGTGGGC-3′) were used for the amplification of IRES-418, while 1b-NS2T7–2487(+) (5′-TAATACGACTCACTATAGGGAGTATGTCGTGTTGCTT-3′) and 1b-NS2–3087(-) (5′-AGCGCGTACGAAGTACGGCA-3′) (11) allowed the PCR amplification of E2/p7/NS2. All high pressure liquid chromatography (HPLC) grade primers were purchased to IBA GmbH. In turn, the IRES-574 fragment (nts 1–574, IRES domains I-VI) was obtained by digestion of pN(1–4728) with the restriction enzyme *Blp*I (NEB). All the DNA fragments were purified by QIAquick Gel Extraction Kit (Qiagen) and eluted in water. These DNA templates (0.5–1 μg) were *in vitro* transcribed using AmpliScribe T7 RNA polymerase (Epicentre) at 37ºC for 4 h. After DNase I treatment, RNA was purified by precipitation with 2.5 M ammonium acetate and 2.5 volumes of absolute ethanol, and finally resuspended in diethylpyrocarbonate (DEPC)-treated water. The integrity of the RNA transcripts was checked in a denaturing 4% polyacrylamide gel, and the concentration of the transcribed RNA was determined by absorbance at 260 nm.

### Control RNA molecule for AFM experiments

The dsRNA molecule used as a control for measuring the height of the A-RNA duplex adsorbed onto mica surfaces modified with (3-aminopropyl)triethoxysilane (APTES) was the 4579-bp-long genomic RNA of the totivirus L-A (57) (kindly provided by Dr. Rosa Esteban, Instituto de Microbiología Bioquímica, CSIC/USAL, Spain), with a theoretical length of 1236 nm.

### RNA folding in different Mg^2+^-containing buffers

RNA transcripts were resuspended at 2–3 nM concentration in folding buffer (100 mM NaCl and 100 mM HEPES pH 7.5) either magnesium-free or supplemented with 2, 4, 6 or 10 mM MgCl_2_. The RNAs were heated at 90ºC for 5 min, cooled on ice for 5 min and incubated at 37ºC for 20 min. The use of other renaturation protocols did not affect the results. Control experiments were performed by incubating IRES-574 in 80% formamide at 90ºC for 5 min, and cooling on ice for 5 min. A folding buffer in which sodium cations were replaced by ammonium ones (100 mM NH_4_Cl, 100 mM HEPES pH 7.5 and 10 mM MgCl_2_) was also used.

### MicroRNA-122 annealing assay

The miR-122 RNA sequence (5′-UGGAGUGUGACAAUGGUGUUUGU-3′) was synthesized and HPLC-purified by IBA GmbH. HCV IRES-574 was folded under different concentrations of Mg^2+^ as described above, incubated (at a concentration of 3 nM) with 15 nM miR-122 at 37°C for 20 min, and the mixture was successively transferred to ice.

### RNA adsorption on mica surfaces

We used APTES-modified mica (58), a method that drives a tight enough immobilization of RNA via electrostatic interactions without damaging RNA molecules. This approach avoided the use of Mg^2+^ or other divalent cations as a bridge to immobilize the negatively charged RNA (59), since they would interfere with our envisaged study. Freshly cleaved muscovite mica Hi-Grade V2 (Monocomp Instrumentation) was treated with a 0.1% solution of APTES (SIGMA-Aldrich) for 15 min, washed with 2-propanol, rinsed with ultrapure, DEPC-treated milliQ water and dried at 37ºC. Then, 30 μl of the RNA sample at 2–3 nM concentration was deposited onto the treated mica disc and incubated in a humidity chamber for 20 min at room temperature (22 ± 2ºC). The RNA-containing mica discs were gently washed with ultrapure, DEPC-treated milliQ water and finally air-dried. A minimum of three independent samples of each HCV IRES preparation (IRES-418, IRES-574 and IRES-574/miR-122 complex) at each buffer composition was imaged.

### AFM imaging

Samples were imaged in air, at room temperature, with two commercial AFM microscopes: PicoSPM (Agilent) and Nanoscope IIIa (Veeco). Their performance was equivalent in the analyzed samples, as assessed after measuring different control preparations of either IRES-574 molecules or IRES-574/miR-122 complexes in parallel using both microscopes (data not shown). Acoustic tapping mode AFM was performed using silicon cantilevers (Nanosensors and Bruker) with spring constants of 0.5–9.5 N/m and resonance frequency in the 50–80 KHz range. Two different kinds of tips, with nominal curvature radius of 2 and 10 nm, were used to obtain different levels of resolution during the image acquisition. The set-points used were kept relatively low, in the 0.3–0.6 V range, in order to use soft imaging conditions. The images (from 512 × 512 to 2048 × 2048 pixels) were recorded at a scan rate of 1 line per second and processed using WSxM v3.1 software (Nanotec) (60). The average length of the RNA molecules was measured using the same software. In the case of branched shapes, the length of every imaged arm of the molecule was measured, and the total length was computed. The number of analyzed IRES molecules in each buffer condition was at least 100 for IRES-574 samples, and at least 50 for IRES-574/miR-122 complexes. The angle between the arms of the selected IRES-574 molecules was measured with Image-J software (61).

### Statistical analysis

The non-parametric Kolmogorov–Smirnov test was used to check the data sets for normality. A non-parametric Mann–Whitney test was applied to IRES-574 at different Mg^2+^ concentration. The independent samples *t*-test was also applied to the IRES-574, IRES-574/miR-122 and E2/p7/NS2 samples incubated under different ionic conditions.

### RNA analysis by native gel electrophoresis

Purified RNA transcripts were internally radiolabeled with [α-^32^P]-GTP (specific radioactivity of 107 dpm/μg) as described (62). After folding under different concentrations of Mg^2+^, the IRES-574 molecule was left on ice for at least 10 min and resuspended at a final 3 nM concentration (3 nM IRES-574 and 15 nM IRES for IRES-574/miR-122 complexes) in loading buffer (previously described in (27)) containing 60% glycerol, 1× TMN, 0.4 μg/μl yeast tRNA (Ambion), 0.4% (w/v) xylene cyanol and 0.4% (w/v) bromophenol blue. The samples were loaded into 0.8-mm thick, non-denaturing 6% polyacrylamide (acrylamide:bis-acrylamide ratio 1:19) gels containing 50 mM Tris-Ac pH 8.3 and 10 mM NH_4_(OAc). The gels were run at 12 mA (constant intensity) for 24 h at 4ºC, and autoradiographed. Product bands were scanned in a Phosphorimager (Storm 820, GE) and quantified with Image Quant 5.2 software (GE). The percentage of formation of each conformer *X* was calculated as follows: % conformer *X* = (signal conformer *X* / Σ signals of all conformers) × 100. Mean values and standard deviations were calculated from three independent experiments.

### RNase T1 and RNase III cleavage assays

Previous studies ((27) and data not shown) showed that the optimal conditions for RNase T1 (Calbiochem) partial digestion of the IRES-574 molecule were 0.001 μg/μl of enzyme (concentrations assayed: 0.01, 0.001, 0.0001, 0.00001 μg/μl), in a 20-min reaction at 37ºC. [^32^P]pCp 3′-end labeled IRES-574 RNA (27) was resuspended in each of the following control conditions: in water and kept on ice for 2 h, to check the RNA integrity (sample ‘I’); in standard folding buffer supplemented with 2 mM ethylenediaminetetraacetic acid (EDTA) (samples ‘0*’ mM Mg^2+^) and in folding buffer without Mg^2+^ (samples ‘0′ mM Mg^2+^), after preheating to 90ºC and slowly cooling to room temperature. RNase T1-digested samples were previously resuspended in the corresponding folding buffer (in the presence of 0–10 mM Mg^2+^), pre-heated to 90ºC for 1 min and cooled to room temperature. Note that 300 cpm of labeled and digested RNA were loaded in each lane. Adequate band separation was achieved when bromophenol blue dye reached 80% of the length of a 7M urea-containing denaturing 4% polyacrylamide gel. IRES-574 molecules incubated with 0.0001 μg/μl RNase T1 under denaturing conditions were used as an electrophoretic mobility control (sample ‘D’). Reference bands were identified from previous studies (10,27). RNase T1 digestion bands that decrease their intensity with increasing Mg^2+^ concentration, consistently detected in different gels and exposure times, were identified.

Commercial *Escherichia coli* RNase III (Ambion), a nuclease specific for dsRNA, was used to cleave the internally labeled (24) HCV-574 molecule. Digestion was performed on renatured HCV RNA under ‘secondary conditions of cleavage’: RNA substrate was pre-heated at 90°C for 1 min, before the addition of reaction buffer [10 mM HEPES–KOH, pH 7.5, 100 mM NH_4_(OAc) and 10 mM Mg(OAc)_2_] and cooled to room temperature. Cleavage reactions were performed at 37°C for 1 h in a volume of 10 μl, in the presence of 20 U RNasin, 0.0005 U/μl (final concentration) of RNase III and 2 μg/μl of yeast tRNA. Note that 300 cpm of digested RNA were loaded in the corresponding gel lane. Digestion products corresponding to fragments spanning nts 1–439 and 27–574 of the IRES-574 molecule, previously characterized by direct RNA fingerprinting analysis and other classical RNA sequencing techniques (24,27), were used as size markers.

## RESULTS

### AFM analysis of HCV IRES in denaturing conditions

A systematic AFM study of HCV IRES molecules adsorbed onto APTES-treated mica surfaces (58) (Supplementary Figure S1) was performed using an RNA transcript encompassing nts 1–574 (domains I–VI) of HCV genome, termed IRES-574 (Figure 1). The theoretical length of the unfolded IRES-574 molecule is 155 nm, assuming that each ribonucleotide unit is 0.27-nm long in an A-RNA helical conformation with a pitch of 3.0 nm and 11 nts per turn. The AFM analysis of the IRES-574 in 80% formamide (Supplementary Figure S2) evidenced that part of its secondary structure is highly stable under harsh denaturing conditions, since the imaged molecules showed 65–80% of the theoretical molecular length. This observation agrees with a previous report on the preservation of secondary structure elements of the hepatitis delta virus ribozyme in 95% formamide (63).

The measured height of the IRES-574 molecule in formamide varied between 1.3 and 2.2 nm (Supplementary Figure S2D). To experimentally check the height of a control linear dsRNA molecule adsorbed onto mica-APTES, we chose the whole genomic RNA of the totivirus L-A (Supplementary Figure S3A). The height of this molecule was 1.2–1.4 nm (Supplementary Figure S3B). Therefore, the height range of the imaged IRES-574 molecule in formamide might correspond to dsRNA regions lying on the surface, together with other elements of non-denatured secondary structure in different orientations.

### AFM analysis of the Mg^2+^-induced IRES-574 folding process

**Figure 2.**
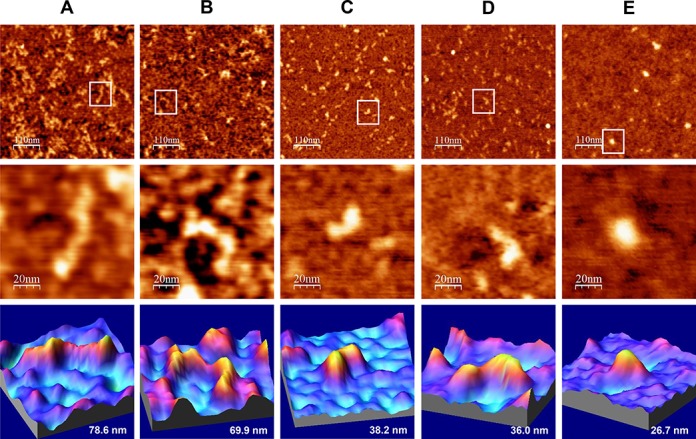
AFM images of IRES-574 molecules in a folding buffer supplemented with increasing Mg^2+^ concentration. IRES-574 RNA molecules were incubated in a buffer containing 100 mM Na^+^, either without Mg^2+^ (panel **A**) or supplemented with 2, 4, 6 and 10 mM Mg^2+^ (panels **B**, **C**, **D** and **E**, respectively). In all panels, top row contains large AFM images of molecules deposited onto a pre-treated mica-APTES surface (550 × 550 nm, scale bar of 110 nm), medium row shows a zoom area containing a selected molecule (100 × 100 nm image, scale bar of 20 nm), and the bottom row depicts a 3D representation of the selected molecule, showing its measured length. The nominal curvature radius of the AFM tips was 2 nm. Overall, this figure shows the Mg^2+^-induced IRES-574 folding process and reveals a major conformational switch when Mg^2+^ concentration increases from 2 to 4 mM.

To analyze the influence of Mg^2+^ on IRES-574 folding, different preparations of the molecule in buffers containing 100 mM Na^+^ supplemented with rising Mg^2+^ concentrations (0, 2, 4, 6 and 10 mM) were visualized by AFM. Tapping mode AFM images of more than 100 molecules under each ionic condition showed that the increase of Mg^2+^ concentration induced conformational changes in IRES-574, while molecular aggregation or dimerization was not observed. Supplementary Figure S4 shows a selection of 12 individual molecules imaged at each Mg^2+^ concentration, and Figure 2 summarizes the observed effect of Mg^2+^ on the conformational rearrangement of the molecule.

IRES-574 molecules incubated in a buffer lacking Mg^2+^ ions showed an elongated, zigzag shape with one to three bumps (Figure 2A and Supplementary Figure S4A), likely corresponding to regions that preserved their secondary structure in the absence of Mg^2+^, in agreement with previous data on minimal HCV IRES (10) and also consistent with a recent analysis of the Mg^2+^-dependent folding of picornavirus IRES (64). At 2 mM Mg^2+^, the imaged shapes showed a large variability even within the same preparation: a majority of elongated morphologies similar to those found at 0 mM Mg^2+^ (Figure 2B) coexisted with some heterogeneous, more compact molecules with 2–4 arms protruding from a central axis (Supplementary Figure S4B) that could inform about certain Mg^2+^-driven tertiary interactions (36,38). A neat increase in the homogeneity and compactness of the imaged molecules was found in the presence of 4 mM Mg^2+^ (Figure 2C and Supplementary Figure S4C). This ionic condition promoted the unexpected formation of compact, comma-shaped molecules in which two arms could be distinguished: a bulky one and a flatter one. Both arms were 15–20 nm long, and the angle between them ranged between 110º and 150º, with a maximum in the interval 117º–127º (Supplementary Figure S5). Similar angles could also be measured in the fraction of non-elongated molecules imaged at 2 mM Mg^2+^ and even in some molecules at 0 mM Mg^2+^ (see examples in Supplementary Figure S4A and B). The trend toward a further RNA compaction was maintained in the presence of 6 mM Mg^2+^ (Figure 2D and Supplementary Figure S4D), with morphologies analogous to those found at 4 mM Mg^2+^ together with some more compact, elliptical-shaped molecules. Finally, in the presence of 10 mM Mg^2+^ only compact molecules with a highly uniform elliptical shape were observed (Figure 2E and Supplementary Figure S4E). Overall, this analysis evidenced that the main structural switch in the IRES-574 conformation occurred between 2 and 4 mM Mg^2+^ concentration.

**Figure 3.**
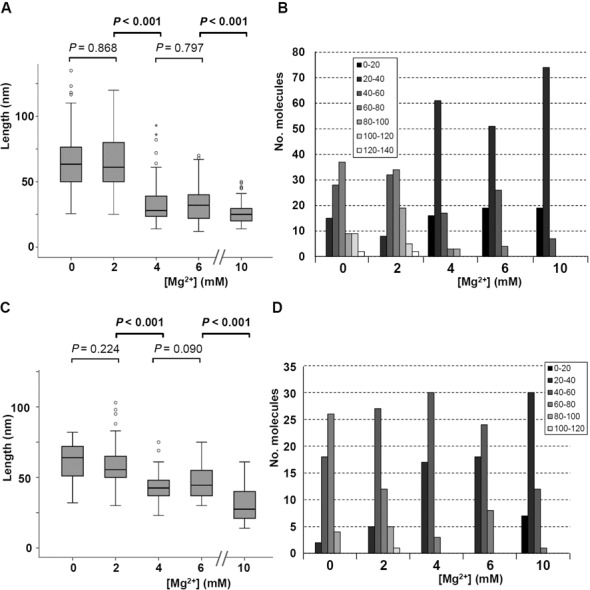
Length distribution of IRES-574 molecules at different Mg^2+^ concentrations. (**A**) Length distribution (computed over 100 molecules) of the IRES-574 molecule in folding buffer supplemented with 0, 2, 4, 6 and 10 mM Mg^2+^ concentration is depicted in a box plot. Boxes represent 25–75 percentile range, vertical lines span 10–90 percentile range and horizontal bar within the box represent the median. Outlier values are depicted as circles (mild outliers) and asterisks (extreme outliers). Statistical *P*-values corresponding to the average length of RNA molecules for 0 versus 2, 2 versus 4, 4 versus 6 and 6 versus 10 Mg^2+^ concentration are shown: statistically significant differences (*P* < 0,001) are marked in bold. (**B**) Distribution of molecular length versus Mg^2+^ concentration, computed for 100 IRES-574 molecules at each buffer composition. Box: intervals of molecular length (nm). (**C**) Length distribution (computed over 50 molecules) of the IRES-574/miR-122 complex at 0, 2, 4, 6 and 10 mM Mg^2+^ concentration depicted in a box plot, as detailed in panel A. (**D**) Distribution of molecular length versus Mg^2+^ concentration, computed for 50 IRES-574/miR-122 complexes in each condition. Box: intervals of molecular length (nm).

To quantitatively study the Mg^2+^-induced folding process, the average end-to-end distance of 100 IRES-574 molecules was measured for each Mg^2+^ concentration (Figure 3). At 0 mM Mg^2+^, molecules with lengths in the range of 25–135 nm were imaged (Figure 3A), most of them being in the interval of 60–80 nm (Figure 3B). Their median length was 63 nm, about 40% of the theoretical length of the molecule. This confirmed that a significant number of RNA secondary structure elements are preserved in the presence of 100 mM Na^+^. IRES-574 molecules under 2 mM Mg^2+^ also showed a broad length distribution between 25 and 120 nm, the majority of them being in the intervals of 40–60 and 60–80 nm. Their median length was 61 nm. A clear shift in the length distribution was found at 4 mM Mg^2+^: the molecules were imaged in a narrower interval of 14–93 nm, with a clear peak in the interval of 20–40 nm and a computed median length of 28 nm. Thus, a greater than 2-fold reduction in the median length was produced when Mg^2+^ concentration increased from 2 to 4 mM, in agreement with the qualitative data derived from Figure 2 and Supplementary Figure S4. In the presence of 6 mM Mg^2+^, the length distribution of the IRES-574 molecules decreased to 12–70 nm while their median length was slightly higher (32 nm). Finally, the additional compaction of the imaged molecules at 10 mM Mg^2+^ was reflected in a very narrow length distribution of the molecules (14–50 nm), concentrated in the interval of 20–40 nm and showing a median length of 25 nm. Along this process, the only statistically significant reductions of the average lengths occurred in the transitions 2–4 and 6–10 mM Mg^2+^ (Figure 3A).

As a control experiment, we analyzed the eventual influence of Mg^2+^ concentration on the folding of a coding region of the HCV genome with a length similar to that of IRES-574: the E2/p7/NS2 junction, spanning nts 2487–3087 of the viral genome (Figure 1A). The results depicted in Supplementary Figure S6 showed that the HCV IRES element, whose biological function relies on its tridimensional structure, is much more dependent on the Mg^2+^ concentration than a coding region of the same length within the same viral genome. We were also interested in analyzing whether two monovalent cations, Na^+^ and NH_4_^+^, might show a differential effect on the folding of IRES-574 molecule in the presence of 10 mM Mg^2+^. The molecules incubated in a buffer containing 100 mM NH_4_^+^ (equivalent to that previously used in RNase III cleavage experiments that supported a ‘closed’ conformation of the HCV IRES including domains I–VI (24)) showed elliptical morphologies that were less compact that those folded in the presence of 100 mM Na^+^ (Supplementary Figure S7). This observation agrees with previous results evidencing that the lower charge density of ammonium with respect to sodium makes the former cation less efficient than the latter one in promoting RNA folding (36,65).

### AFM analysis of a 418-nt-long HCV IRES transcript in the presence of 4 mM Mg^2+^

**Figure 4.**
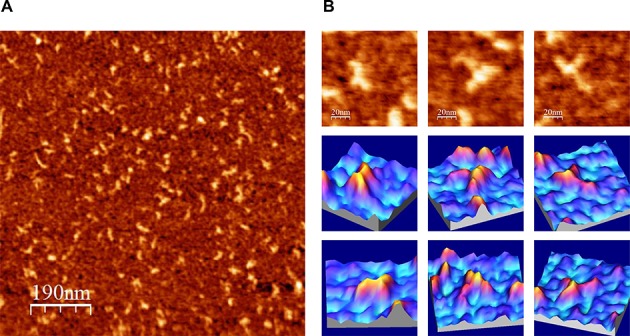
AFM images of IRES-418 molecules in a folding buffer containing 4 mM Mg^2+^. (**A**) Large AFM image (1000 × 1000 nm) of molecules deposited onto a mica-APTES surface. (**B**) AFM images (100 × 100 nm) of three selected molecules (top row) and their corresponding 3D representations from two view angles (medium and bottom rows). The nominal curvature radius of the AFM tips was 2 nm.

We also analyzed by AFM an RNA molecule spanning nts 1–418 of HCV genome (molecule termed IRES-418). It contained the IRES domains II–IV flanked upstream by domain I and, downstream, by the first 76 nts of the core coding region (Figure 1). Most of the imaged IRES-418 molecules in the presence of 4 mM Mg^2+^ showed elongated conformations together with branched shapes where three arms of different lengths protruded from a central axis, some of which exhibited a characteristic ‘λ-like’ shape (Figure 4).

### Monitoring the Mg^2+^-induced folding of IRES-574 by gel-shift

**Figure 5.**
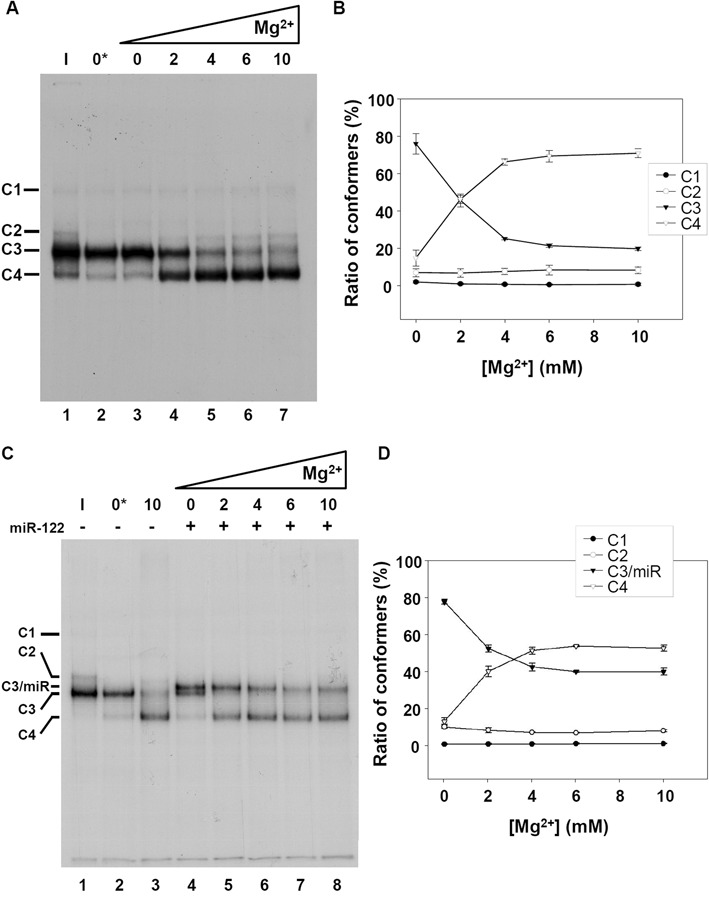
Native gel electrophoresis of the HCV IRES-574 at different Mg^2+^ concentrations. (**A**) Autoradiography of a non-denaturing 6% polyacrylamide gel. Internally labeled IRES-574 RNA was resuspended at 3 nM in each of the following conditions: in water and kept on ice for 10 min, as a control of intact RNA (sample ‘I’, lane 1); in standard folding buffer supplemented with 2 mM EDTA (sample ‘0*’ mM Mg^2+^, lane 2); in folding buffer alone (sample ‘0′ mM Mg^2+^, lane 3); in folding buffer containing Mg^2+^ at a final concentration of 2, 4, 6 and 10 mM (lanes 4–7). Resuspended RNA in lanes 2–7 was pre-heated to 90ºC for 1 min and slowly cooled down to room temperature. Four conformers, labeled as C1 to C4, are detected at different ratios in all the lanes. (**B**) Quantification of the percentage of formation of each conformer at 0, 2, 4, 6 and 10 mM Mg^2+^ in three independent experiments analogous to that showed in panel A (lanes 3–7). Mean values and standard deviations are shown. (**C**) Same as panel A showing the effect of the interaction of miR-122 with IRES-574: ice-incubated control IRES-574 RNA (sample ‘I’, lane 1); IRES-574 alone in buffer supplemented with EDTA (sample ‘0*’ mM Mg^2+^, lane 2); IRES-574 in folding buffer containing Mg^2+^ at 10 mM concentration (lane 3); IRES-574 (at 3 nM concentration) incubated with miR-122 (at 15 nM concentration) in folding buffer either lacking Mg^2+^ (lane 4) or in the presence of increasing concentrations of Mg^2+^ from 2 to 10 mM (lanes 5–8). Five conformers (labeled as C1, C2, C3/miR, C3 and C4) were observed in total. (**D**) Quantification of the percentage of formation of conformers C1, C2, C3/miR and C4 at 0–10 mM Mg^2+^ in three independent experiments analogous to that showed in panel C (lanes 4–8). Mean values and standard deviations are shown.

To deepen into the conformational rearrangements imaged by AFM in the IRES-574 molecules, two complementary techniques for RNA structural analysis were used: gel-shift and ssRNA-specific digestions. Native gel electrophoresis allowed the identification of only four major bands, corresponding to conformers termed C1 to C4 (Figure 5A). Among them, C1 and C2 were minority under all the buffer conditions assayed, while the fast-migrating conformers C3 and C4 were the most abundant. The band intensity was quantified in three independent experiments, and the ratio of each conformer was graphically represented (Figure 5B). In the absence of Mg^2+^ (both with and without added EDTA, see Materials and Methods) the most intense band was C3 (mean of 71.7% and 76.1% in lanes 2 and 3 of Figure 5A, respectively), followed by C4 (18.3% and 14.8%, respectively). Thus, the use of EDTA to remove the residual Mg^2+^ ions eventually trapped into the IRES-574 molecule showed a very limited effect in our experimental conditions. In turn, when the folding buffer contained 2 mM Mg^2+^ a reduction of the amount of C3 was observed concomitantly to the increase of C4, their relative intensities equalizing (45.7% and 46.3%, respectively). The increase of Mg^2+^ up to 4 mM completed the observed shift between C3 and C4 (rates of 25.3% and 66.3%, respectively). A further increase of Mg^2+^ up to 6 mM produced slight variations (21.5% of C3 and 69.4% of C4) while the dominance of the most compact conformer was again reinforced at 10 mM Mg^2+^ (19.9% of C3 and 71.0% of C4).

### Analysis of the folding process of IRES-574 by partial RNase T1 cleavage

**Figure 6.**
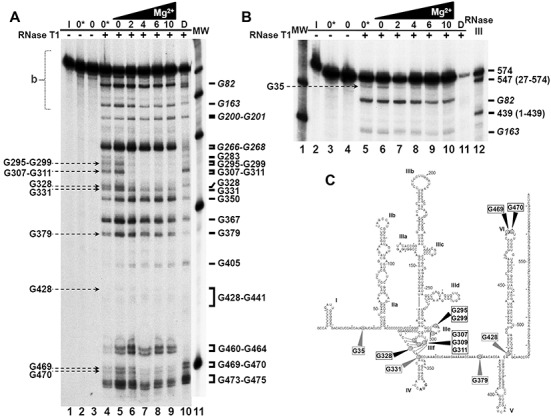
Mg^2+^-induced protection from RNase T1 cleavage. (**A**) Partial RNase T1 cleavage of 3′-end labeled IRES-574 resolved in a denaturing gel. Undigested control samples (lanes 1–3) are described in the Materials and Methods section. Lanes 4–9 correspond to samples resuspended in folding buffer either without Mg^2+^ (lanes 4 and 5) or supplemented with 2, 4, 6 and 10 mM Mg^2+^ (lanes 6–9, respectively), renatured and digested by RNase T1. Electrophoretic mobility control: sample ‘D’ (see Materials and Methods), lane 10. Molecular weight (100–500 nt) markers (‘MW’) corresponding to a less exposed gel were added as lane 11. Reference bands are shown on the right of the gel. Bands that decrease their intensity with increasing Mg^2+^ are identified by arrows on the left. (**B**) A longer electrophoretic run used to resolve the upper region marked with ‘b’ in panel A. MW markers are shown in lane 1. Lanes 2–11 correspond to lanes 1–10 in panel A. Control RNase III digestion fragments spanning nts 1–439 and 27–574 of the IRES-574 molecule are shown in lane 12. Reference positions 82 and 163 (10) are also marked. The band corresponding to nt G35, which decreases its intensity with increasing Mg^2+^ concentration, is identified on the left. (**C**) Secondary structure of the HCV IRES-574 molecule, showing the positions of the RNase T1 cleavage sites corresponding to the bands identified in panels A and B. Nucleotides that either decrease their intensity or virtually disappear with increasing Mg^2+^ concentration are marked with grey and black arrowheads, respectively.

Partial RNase T1 cleavage assays were carried out on IRES-574 molecules under different Mg^2+^ concentrations. Nuclease T1 cuts after G nts in ssRNA regions, being a sensitive method to detect global rearrangements in RNA structure that introduce steric hindrance. Since the Mg^2+^-dependent IRES structure has been previously analyzed for domains II and III in the context of a 332-nt-long molecule (10), we focused our study on the effect of Mg^2+^ on the 574-long transcript that also included domains I, IV, V and VI. The partial digestion of IRES-574 molecules (Figure 6) showed a defined cleavage pattern in the absence of added divalent ions, which changed as a result of the presence of increasing Mg^2+^ concentrations. The samples without Mg^2+^ (lanes 4 and 5 of Figure 6A) showed that the cleavage yield of the EDTA-treated molecule (lane 4) was slightly lower than that of the sample without EDTA (lane 5). Thus, it is likely that the total absence of Mg^2+^ ions in the EDTA-containing buffer impaired the activity of the nuclease. Upon addition of Mg^2+^, the overall reactivity of IRES-574 decreased (lanes 6–9), thus reflecting a progressive Mg^2+^-induced compaction of the molecule, in agreement with previous results (10). However, several regions of the molecule, in particular, certain hairpin loops, remained accessible to the nuclease.

We chose the electrophoretic bands that could be used as a reference to identify the nts whose reactivity to RNase T1 cleavage was affected by the Mg^2+^ concentration. Among them, positions 82, 163, 200–201 and 266–268 corresponded to the highly accessible Gs located at the apical loops of subdomains IIb, IIIa, IIIb and IIId, respectively (10), while reactive positions between nts 350 and 475 were detected in other previous work (27) (Figure 6A). Additional reference bands were identified as the eventually accessible Gs between positions 268 and 350: 283, 295, 299, 307, 309, 311, 328 and 331 (the latter G was previously shown to be a reactive nucleotide despite being paired in the secondary structure models (10)). This allowed us to identify the main Gs that changed their reactivity to RNase T1 as a result of the Mg^2+^-driven compaction of the IRES-574 molecule. Additionally, a longer electrophoretic run allowed resolving the upper part of the gel showed at Figure 6A, corresponding to the 5′-end of the HCV IRES (Figure 6B).

The first observation was that the region encompassing nts 379–460 showed a very low reactivity to RNase T1 even in the absence of Mg^2+^, which is indicative of the high stability of the RNA stems present at domains V and VI, as well as the protection of two Gs (at positions 435 and 436) whose accessibility might have been predicted from the secondary structure of the HCV IRES (Figure 1). This exemplifies that in the absence of added Mg^2+^ certain elements of secondary structure are stabilized by the monovalent cations present in the buffer, as imaged by AFM. On the contrary, certain nts in the analyzed region (including G350, G367, G460-G464 and G473-G475) remain highly reactive to RNase T1 digestion in folding buffers containing Mg^2+^ concentrations in the range 0–10 mM.

The conformational changes induced by Mg^2+^ (lanes 5–9 of Figure 6A, and lanes 6–10 of Figure 6B) were accompanied by a decrease in the intensity of at least nine RNase T1 bands. This effect was detected when Mg^2+^ concentration increased from 0 to 2 mM (bands G295-G299, G307-G309-G311, G328, G428, G469 and G470 in Figure 6A; G35 in Figure 6B) and from 2 to 4 mM (G331 and G379, Figure 6A). Among them, the most pronounced protections from RNase T1 cleavage was found in bands G295-G299, G307-G309-G311, G328, G469 and G470. In all the positions showing Mg^2+^-dependent reactivity, the band intensities were similar at 4, 6 and 10 mM Mg^2+^, what suggests that the main conformational rearrangement of the IRES-574 molecule is already completed at 4 mM Mg^2+^, in agreement with our previous AFM and gel-shift results. The nts whose reactivity to RNase T1 is affected by Mg^2+^ are located at the spacer region between domains I and II (G35), the IIIef/IV pseudo-knot (G295-G299, G307-G309-G311 and G328), the basal region of domain IV (G331), the spacer between domains IV and V (G379), the basis of domain VI (G428) and the apical loop of this domain (G469 and G470), as depicted in Figure 6C.

### Effect of miR-122 on the Mg^2+^-induced conformational switch of IRES-574

The microRNA miR-122 interacts with two tandem binding sites in the I-II spacer region of the HCV IRES (25,26) (Figure 1) and induce switching of the IRES element in its natural sequence context (domains I–VI) between two alternative conformers: from a quick migrating form associated with a ‘closed’ conformation to a slow migrating one corresponding to an ‘open’ conformation) (27). Thus, the possibility that the conformational effect of Mg^2+^ on IRES-574 folding was inversely related to that of miR-122 deserved a detailed analysis by AFM and gel-shift. First, IRES-574 at 3 nM concentration was titrated using native gel electrophoresis with a range of miR-122 concentrations (from 0.5 to 150 nM) in the folding buffer containing 6 mM Mg^2+^. As a 15 nM concentration of miR-122 was found to initiate the linear phase of the saturation curve from ‘closed’ to ‘open’ conformations (data not shown), such conditions (corresponding to an IRES-574:miR-122 ratio of 1:5) were used for subsequent AFM and gel-shift experiments.

**Figure 7.**
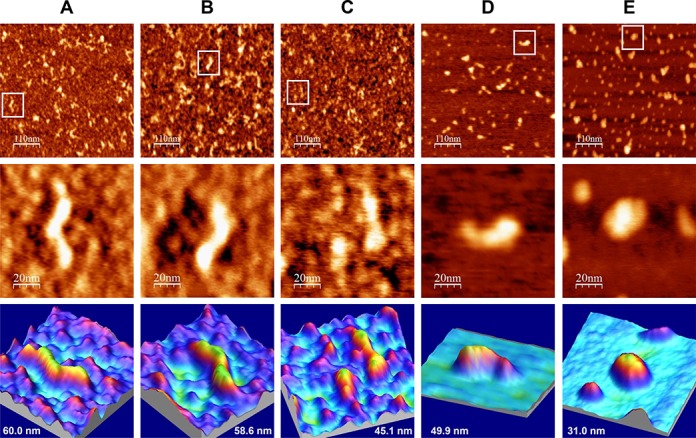
AFM images of IRES-574/miR-122 complexes in a folding buffer supplemented with increasing Mg^2+^ concentration. IRES-574 RNA molecules were folded in a buffer containing 100 mM Na^+^, either without Mg^2+^ (panel **A**) or supplemented with 2, 4, 6 and 10 mM Mg^2+^ (panels **B**, **C**, **D** and **E**, respectively) and incubated with miR-122 (see Materials and Methods). Large AFM images (550 × 550 nm, top row), zoom areas containing a selected molecule (100 × 100 nm images, medium row) and 3D representations of the selected molecule showing its measured length (bottom row) are shown. The medium and bottom row at panel C show an open and a closed IRES-574 conformer coexisting at 4 mM Mg^2+^, the depicted length corresponding to the open one. Two miR-122 molecules are imaged at panel E (10 mM Mg^2+^) together with a compact IRES-574 conformer. The nominal curvature radius of the AFM tips was 2 nm.

The visualization by AFM of IRES-574/miR-122 complexes in buffers containing 100 mM Na^+^ and 0–10 mM Mg^2+^ was performed as described above. As a control experiment, the miR-122 molecule alone (whose 23-nt-long sequence corresponds to a theoretical length of 6.2 nm) was imaged at 15 nM concentration under different ionic conditions, showing a uniform and elliptical shape of 10–14 nm in length (Supplementary Figure S8). Therefore, unbound miR-122 and IRES-574 molecules were clearly distinguishable in the analyzed AFM images (see examples at Figure 7E and Supplementary Figure S9), while neither dimerization nor molecular aggregation were observed.

AFM images of IRES-574/miR-122 complexes in the folding buffer lacking Mg^2+^ ions showed a majority of elongated and angular shapes similar to those previously found in the absence of miR-122 (Figure 7A and Supplementary Figure S9A). Their median length (computed over 50 IRES-574 molecules) was 64 nm (Figure 3C), and most of the imaged molecules were in the length interval of 60–80 nm (Figure 3D). Elongated and zigzaging molecules were also imaged at 2 mM Mg^2+^ (Figure 7B and Supplementary Figure S9B), with a median length of 56 nm (Figure 3C) and the majority of them being 40–60 nm long (Figure 3D). In contrast to the observation in the absence of miR-122, the complex IRES-574/miR-122 at 4 mM Mg^2+^ did not show uniform, comma-shaped images, but a heterogeneous distribution of partially elongated and branched shapes together with a fraction of more compact ones (Figure 7C and Supplementary Figure S9C). The majority of their molecular lengths belonged to the interval of 40–60 nm, with a computed median value of 43 nm (Figure 3C and D). Thus, the decrease in the median length associated to the transition from 2 to 4 mM Mg^2+^ was 13 nm (23% of the length at 2 mM), while the previously observed shortening in the absence of miR-122 (Figure 3A) was 33 nm (54% of the length at 2 mM). However, both decreases showed statistically significant differences at the level of the average molecular length. The coexistence of partially elongated and compact shapes was also imaged at 6 mM Mg^2+^ (Figure 7D and Supplementary Figure S9D): most of the molecular lengths were still found in the interval of 40–60 nm, with a median value of 45 nm (Figure 3C and D). Among the molecules observed in the presence of 2, 4 and 6 mM Mg^2+^, examples with 3 to 4 arms, analogous to those found in the absence of miR-122 at 2 mM Mg^2+^, were imaged, as well as additional morphologies including triangles, F- and C-shaped molecules (Supplementary Figure S9B–D). Finally, the maximum Mg^2+^ concentration assayed (10 mM) promoted a clear and statistically supported compaction of the imaged molecules to a median length of 28 nm (Figure 3C and D), with a majority of 20–40 nm long structures (Figure 3D) showing triangular, elliptical and round shapes (Figure 7E and Supplementary Figure S9E).

The effect on IRES-574 conformation of increasing Mg^2+^ concentration from 0 to 10 mM in the presence of miR-122 was also analyzed by gel-shift (Figure 5C and D) as described above, and the results were compared to those previously obtained in the absence of miR-122 (Figure 5A and B). In the absence of miR-122 and Mg^2+^ (lane 2 of Figure 5C, equivalent to lane 2 of Figure 5A) a single band, corresponding to the slow migrating form (conformer C3), was observed. The addition of miR-122 (lane 4 of Figure 5C), displaced more than one half of the total signal to a slightly slower migrating band, which corresponded to the IRES-574/miR-122 complex (conformer termed ‘C3/miR’), leaving a residual band with an electrophoretic mobility equivalent to that of the unbound IRES-574 molecule (C3). The presence of 2 mM Mg^2+^ in the folding buffer (lane 5 of Figure 5C) virtually eliminated the fraction of unbound IRES-574, thus showing that the divalent ion promoted the annealing of miR-122 (present in a 5:1 excess) with the available IRES-574 molecules. The relative band intensity of the conformer C3/miR (quantified in three independent experiments and graphically represented in Figure 5D) showed a mean value of 52.1% of the total signal. Also, a fast migrating band (conformer C4) increased its mean ratio from 12.6% in the absence of Mg^2+^ to 39.7% at 2 mM Mg^2+^. Thus, the Mg^2+^-driven promotion of a ‘closed’ conformation of IRES-574 occurred in a smaller fraction of molecules with respect to the previous observation in the absence of miR-122 (see Figure 5B). The further addition of Mg^2+^ increased the mean ratio of C4 versus C3/miR conformers to 51.0%:42.1%, 53.4%:39.6% and 52.3%:39.5% at 4, 6 and 10 mM Mg^2+^, respectively (Figure 5D). Therefore, two main differences were observed with respect to the situation found in the absence of miR-122 (compare Figure 5B and D): (i) when miR-122 was present in the medium, the Mg^2+^ concentration at which the two major conformers (C3/miR and C4) reached an equal representation was 3 mM, a value higher than that for the equilibrium between C3 and C4 in the absence of miR-122 (2 mM Mg^2+^); (ii) miR-122 affected the equilibrium between ‘open’ and ‘closed’ conformations at 4–10 mM Mg^2+^ in such a way that the ‘closed’ conformer was still predominant over the ‘open’ one, though to a lesser extent than in the absence of the microRNA (roughly a 5:4 ratio versus a 7:2 ratio).

## DISCUSSION

In this work, we used AFM for visualizing the Mg^2+^-induced conformational rearrangement of a 574-nt-long RNA molecule that includes the HCV IRES element together with its flanking domains I, V and VI. The quantitative analysis of the molecular lengths measured at each Mg^2+^ concentration revealed a two-regime behavior in the IRES-574 folding. A sharp conformational switch was monitored from a heterogeneous distribution of elongated shapes at 0–2 mM Mg^2+^ to a uniform population of compact structures at 4–6–10 mM Mg^2+^. It is reasonable to assume that the alternative conformations imaged at 2 mM Mg^2+^ reflect the relative movements of rigid parts (defined by stable secondary structures) instead of the presence of disordered regions, in agreement with previous analysis of the IRES domains II–IV at 2.5 mM Mg^2+^ (19). Thus, a fraction of the imaged shapes at 2 mM Mg^2+^ could allow the identification of some of the folded, individual IRES domains, while the morphologies imaged at 4 and 6 mM Mg^2+^ would eventually reveal long-range RNA interactions among the preformed domains. In our system, 2 mM Mg^2+^ could represent the divalent cation concentration at which some compact or ‘collapsed’ RNA intermediate conformations appear before the proper tertiary structure is acquired, by analogy to certain group I introns where the transition from collapsed RNA conformations to their functional tertiary structures occurs at 2–3 mM Mg^2+^ (66).

Gel-shift analysis confirmed the existence of a switch between two major, alternative conformations of the IRES-574 molecule. The stepwise increase of Mg^2+^ concentration from 0 to 4 mM completely shifted the ratio between such RNA conformers toward the most compact one, both of them being equally represented at 2 mM Mg^2+^. A previous study showed that the HCV IRES containing domains II–IV folded into a unique, extended structure with certain degree of flexibility at 2.5 mM Mg^2+^ (10). This fact suggests that the presence of two major conformers in the molecule IRES-574 at 2 mM Mg^2+^ is likely due to the conformational flexibility allowed by the flanking domains I, V and VI.

**Figure 8.**
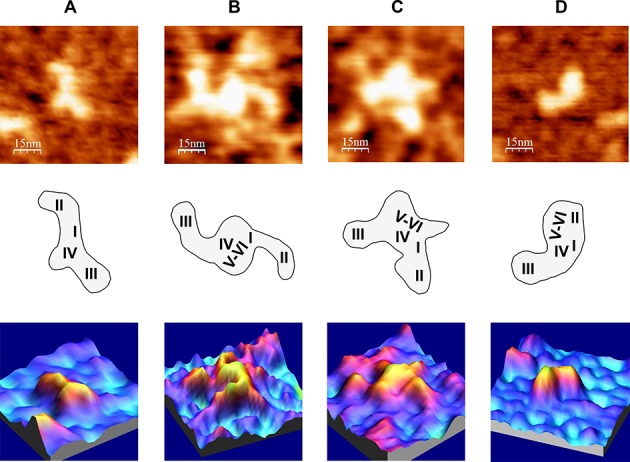
AFM images of HCV IRES-containing RNA molecules under different ionic conditions, in the absence of miR-122. (**A**) IRES-418 molecule at 4 mM Mg^2+^. (**B**) and (**C**) Two examples of IRES-574 molecules at 2 mM Mg^2+^. (**D**) IRES-574 molecule at 4 mM Mg^2+^. AFM images (75 × 75 nm) of the selected molecule (top row), schematic representations of the molecule sowing the suggested assignment of IRES domains (medium row) and 3D representations of the selected molecules (bottom row) are shown. The nominal curvature radius of the AFM tips was 2 nm.

The AFM analysis of the shorter transcript IRES-418 (containing domains I–IV) showed that this molecule can adopt three-arms morphologies at 4 mM Mg^2+^, including λ-shaped conformations. These molecular shapes are consistent with those derived from a previous TEM analysis (17) of the HCV IRES (nts 1–380) at 5 mM Mg^2+^, and from a SAXS-based model (19) of a shorter version of the HCV IRES (nts 39–371) at 2.5 mM Mg^2+^. Both approaches showed molecules with three main arms protruding from a central axis, what has been interpreted as a structure based on a flexible pseudo-knot that constitutes a hinge between IRES domains II, III and IV (14). The comparison of our AFM images with such previous data allowed us to hypothesize that the IRES domains II–IV might be assigned as depicted in Figure 8A. Hence, the bended arm corresponding to the upper part of the λ-like imaged shapes (showing a characteristic angular morphology previously revealed by NMR (13), also identified in a SAXS-based model of the HCV IRES (19) and in the cryo-EM map of the HCV IRES bound to the 40S ribosomal subunit (18)) would correspond to domain II. In turn, the bulky and straight arm would contain the apical part of domain III (subdomains IIIabc (16)). The comparison of our data with the SAXS-based model (19) also suggests that the third, shorter arm imaged by AFM could be assigned to either subdomain IIId or domain IV plus the core-coding sequence fragment. The putative domain I cannot be identified by AFM in any of the molecules, however, it is assumed to be placed next to the basal region of domain II.

Although it is evident that further experiments will be needed to unambiguously identify individual IRES domains in the molecules imaged by AFM, the three-arms conformation of the IRES-418 at 4 mM Mg^2+^ helped us interpret a fraction of the AFM images of the IRES-574 molecule at 2 mM Mg^2+^, in which three or four arms have been imaged (two representative examples are shown in Figure 8B and C). According to our model, domains II and III could be distinguished, while the downstream domains V-VI might either be placed in the central region of the molecule close to the pseudo-knot (Figure 8B) or (thanks to the flexibility provided by the ssRNA spacer between domains IV and V) partially protrude from such central region as one or two extra arms (Figure 8C). In turn, the compact two-arms, comma-shaped morphology showed by most of the IRES-574 molecules at 4 and 6 mM Mg^2+^ (Figure 8D) would suggest the presence of a long-range interaction of the downstream domains V-VI with either domains I-II or domain III. The comparison of the images in Figure 8 and Supplementary Figure S4 with previously published data (17,19) would favor the former possibility. Therefore, we hypothesize that, in the IRES-574 molecule, the downstream domains V (likely excluding its apical region, which is also present in the IRES-418 molecule, see Figure 1) and/or VI could interact with the arm composed of domains I-II provided that Mg^2+^ concentration is at least 2 mM, this interaction being favored at 4 mM Mg^2+^.

In order to test this hypothesis, a complementary analysis of the IRES-574 molecule was performed by RNase T1 probing. Our results showed that the main transition in the observed conformational rearrangements was produced between 0 and 4 mM Mg^2+^ concentration: the intensity of at least nine RNase T1 bands (corresponding to 12 reactive Gs) decreased, and such a protection was already evident at 2 mM Mg^2+^ in seven bands (10 Gs). The protected positions at 2 mM Mg^2+^ included six Gs directly involved in the formation of the IIIef/IV pseudo-knot (14). The requirement of at least 0.25 mM Mg^2+^ for the establishment of this key tertiary interaction has been shown in the HCV IRES containing domains II–IV (10). Therefore, it seems evident that the formation of the central pseudo-knot is not affected by the presence of the flanking domains I, V and VI. Among the remaining six protected Gs, two of them (G35 and G428) are placed at the I-II spacer region and at the basis of domain VI, respectively. Notably, they lie at both strands of a previously proposed long-range annealing motif (involving sequences at nts 24–38 and 428–442, Figure 1) that could promote a ‘closed’ conformation of HCV IRES in its natural sequence context (22–24). This fact suggested that such a long-range RNA interaction could be involved in the Mg^2+^-induced conformational switch reported in IRES-574.

The conformational rearrangement of the IRES-574 molecule produced between 0 and 4 mM Mg^2+^ (both conformers being in equilibrium at 2 mM Mg^2+^) could rely, first, on the formation of the IIIef/IV pseudo-knot, which affects the central region of the IRES element including the basis of domain IV. Concomitantly, this tertiary interaction would allow the long-range RNA interaction involving domains I-II and domains V-VI. In the model we are envisaging, such interactions could be responsible for the main conformers visualized by AFM and quantified by gel-shift analysis: (i) elongated molecules at 0 mM Mg^2+^, where only intra-domain, stable secondary structure elements are present; (ii) molecules with heterogeneous and angular shapes at 2 mM Mg^2+^, including topologies with three arms, likely corresponding to domains I-II, III and V-VI, which protrude from a central region defined by the IIIef/IV pseudo-knot; (iii) homogeneous, comma-shaped molecules at 4–6 mM Mg^2+^ showing two arms, the first of them being likely composed of the interacting domains (I-II) + (V-VI) and the second one containing domain III alone.

The angle formed between the two IRES-574 arms at 4 mM Mg^2+^ showed a maximum in the interval of 117º–127º. Previous studies indicated that the dominant motion in HCV IRES is the in-plane movement of domains II and III toward and away from each other (19). Thus, it is reasonable to assume that, while in the IRES molecule than contains domains II–IV imaged by EM (17) the maximum angle between domains II and III lies in the interval 100º–110º, the presence in IRES-574 of a bulky arm composed of the interacting domains (I-II) + (V-VI) displaces the maximum in the distribution of the angles formed with domain III toward higher values.

The AFM and gel-shift analyses performed in the presence of miR-122 supported the involvement of the miR-122-binding site at the I-II spacer region in the reported molecular switch. AFM images showed that, under Mg^2+^ concentrations of 0 and 10 mM Mg^2+^, the distribution of molecular lengths and shapes was similar in IRES-574 molecules and in IRES-574/miR-122 complexes. However, the sharp conformational switch imaged in IRES-574 molecules when Mg^2+^ concentration increased from 2 to 4 mM was not evident in the presence of miR-122, since the IRES-574/miR-122 complex folded in heterogeneous shapes of different lengths in the interval of 2–6 mM Mg^2+^ concentration. The results of gel-shift analyses were in agreement with the equilibrium between ‘open’ and ‘closed’ conformations of the IRES-574/miR-122 complex in buffers containing Mg^2+^ concentrations higher than 2 mM.

Previously, a transition from ‘closed’ to ‘open’ IRES-574 conformation was demonstrated in the presence of miR-122 (27). Here, we show that this transition can also be promoted by Mg^2+^, though in the opposite direction: while miR-122 favours the ‘open’ form, the increasing Mg^2+^ displaces the equilibrium toward the ‘closed’ conformer. Remarkably, both effectors have been described to affect HCV IRES-dependent initiation of translation *in vitro*: the presence of miR-122 stimulates the process (31), while the increase of Mg^2+^ concentration up to 5 mM allows IRES-dependent translation initiation in the absence of initiation factors (40). Therefore, in the frame of the many regulated functions in which the interactions between miR-122 and HCV IRES may participate (32–35), the data reported here point toward the existence of a bidirectional conformational switch stimulated (in opposite directions) by two effectors of very different nature.

Gel-shift analysis showed that ‘open’ and ‘closed’ conformations of IRES-574 are in equilibrium at 2 mM Mg^2+^ in the absence of miR-122, and at 3 mM Mg^2+^ when the micro-RNA is present in the medium. These values are higher than the closely regulated 0.5–1 mM concentration (0.59 mM in rat hepatocytes) of free Mg^2+^ in the eukaryotic cytoplasm (67–69), where positively charged RNA-binding proteins and other effectors also modulate the functional structure of HCV IRES. However, such a 2–3 mM Mg^2+^ concentrations correspond to the 2.5 mM value at which HCV IRES can promote the initiation of translation in the absence of the complete set of initiation factors (40) and define the upper limit of the Mg^2+^ concentration that allows IRES activity in translation-competent extracts (2.5 mM (39)). Therefore, the Mg^2+^-induced ‘open’/’closed’ switch reported here could be of relevance for IRES-dependent translation initiation *in vitro* under different experimental settings. Our results also support that the complementary sequences present at the I-II spacer and at the basal region of domain VI are prone to establish a long-range RNA interaction provided that the ionic or biochemical environment of the folded HCV 5′ UTR allows a compatible tertiary structure. The structural data reported here provide further insights on the conformational plasticity of HCV IRES in its genomic context (22–24) and, as a result, encourage the development of novel HCV inhibitors based on small molecules targeting either the IRES regions essential for translation initiation (2,3) or the liver-specific microRNAs that act as enhancers of viral replication (70).

In summary, here we report for the first time the use of AFM to analyze the 3D structure of an IRES element. We have shown that, in its natural sequence context, HCV IRES can undergo a Mg^2+^-induced molecular switch between two alternative conformations, such a structural rearrangement being hindered by the presence of miR-122 in the medium. Our data are compatible with those previously reported for HCV IRES domains II–IV based on chemical and enzymatic probing, EM and SAXS-derived models. Additionally, our results shed light on the 3D conformation of HCV IRES in its natural sequence context, thus reinforcing the previously suggested structural continuity between the domain I, the minimal HCV IRES element and (despite the apparent functional discontinuity between coding and non-coding sequences) the essential domains V and VI within the core coding region.

## SUPPLEMENTARY DATA

Supplementary Data are available at NAR Online.

SUPPLEMENTARY DATA
