# Supplementary material for: A magnesium-induced RNA conformational switch at the internal ribosome entry site of hepatitis C virus genome visualized by atomic force microscopy
Source: Nucleic Acids Res. 2014 Dec 15;43(1):565–80. doi: 10.1093/nar/gku1299 (PMC4288189; doi:10.1093/nar/gku1299)
Supplement: SUPPLEMENTARY DATA [file supp_43_1_565__index.html]

A magnesium-induced RNA conformational switch at the internal ribosome entry site of hepatitis C virus genome visualized by atomic force microscopy — SUPPLEMENTARY DATA 

# A magnesium-induced RNA conformational switch at the internal ribosome entry site of hepatitis C virus genome visualized by atomic force microscopy

## SUPPLEMENTARY DATA

**Files in this Data Supplement:**

- SUPPLEMENTARY DATA
