# Supplementary material for: A magnesium-induced RNA conformational switch at the internal ribosome entry site of hepatitis C virus genome visualized by atomic force microscopy
Source: Nucleic Acids Res. 2014 Dec 15;43(1):565–80. doi: 10.1093/nar/gku1299 (PMC4288189; doi:10.1093/nar/gku1299)
Supplement: SUPPLEMENTARY DATA [file supp_gku1299_nar-03086-r-2014-File012.pdf]

A magnesium-induced RNA conformational switch at the internal ribosome entry site of hepatitis C virus genome visualized by atomic force microscopy

Ana García-Sacristán[1,2], Miguel Moreno[1,‖], Ascensión Ariza-Mateos[2,3,‖], Elena López-Camacho[1,4], Rosa M. Jáudenes[1], Luis Vázquez[4], Jordi Gómez[2,3], José Ángel Martín-Gago[1,4] and Carlos Briones[1,2,*].

[1] Department of Molecular Evolution, Centro de Astrobiología (CSIC-INTA). Torrejón de Ardoz, Madrid, 28850, Spain.

[2] Centro de Investigaciones Biomédicas en Red de Enfermedades Hepáticas y Digestivas. (CIBERehd), Spain.

[3] Laboratory of RNA Archaeology, Instituto de Parasitología y Biomedicina "López-Neyra" (CSIC), Parque Tecnológico Ciencias de la Salud, Armilla, Granada, 18016, Spain.

[4] Instituto de Ciencia de Materiales de Madrid (CSIC). Cantoblanco, Madrid, 28049, Spain.

[‖] M.M. and A.A-M. contributed equally to this work.

* To whom correspondence should be addressed: Carlos Briones; Tel: +34 91 5206411; Fax: +34 91 5201074; E-mail: cbriones@cab.inta-csic.es

Supplementary Data

Supplementary Figure 1

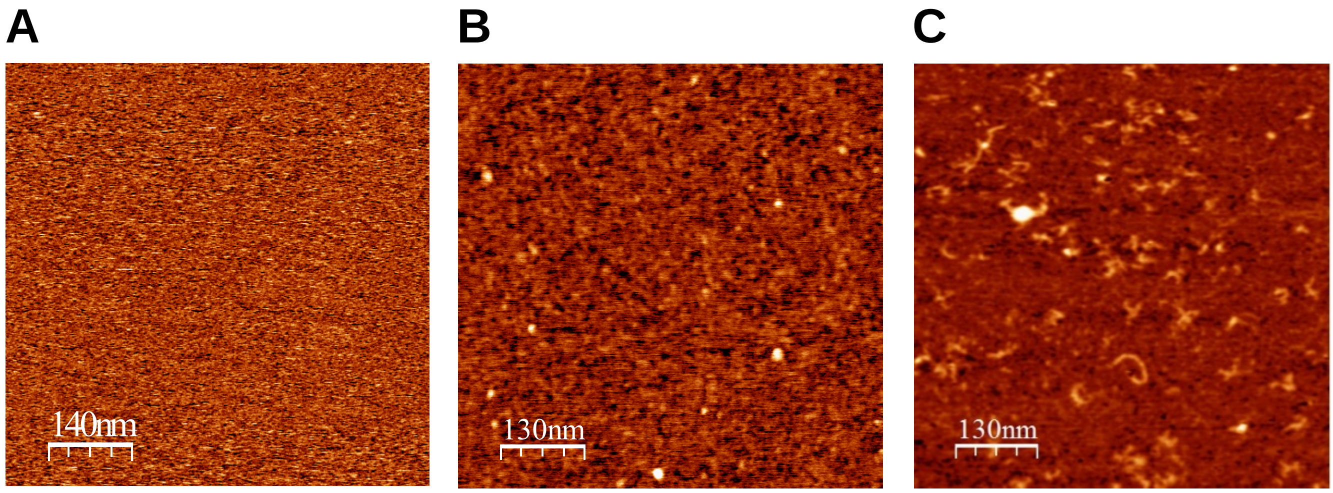

Supplementary Figure 1. Schematic representation of the adsorption process of RNA molecules onto (3-aminopropyl)triethoxysilane (APTES)-treated mica. (A) Freshly cleaved muscovite mica exposes a flat surface. **(B)** After treatment with APTES, the mica surface becomes positively charged. AFM images of mica-APTES surface show a root mean square (rms) roughness smaller than 0.65 nm, a low background value which allows distinguishing any biomolecule adsorbed onto the surface. **(C)** RNA deposited onto the mica-APTES surface interacts electrostatically with the charged surface. Preliminary experiments were carried out to check that the RNA preparation and AFM imaging did not damage or degrade the RNA molecules (data not shown). The nominal curvature radius of the AFM tips used for producing these images was 2 nm.

Supplementary Figure 2

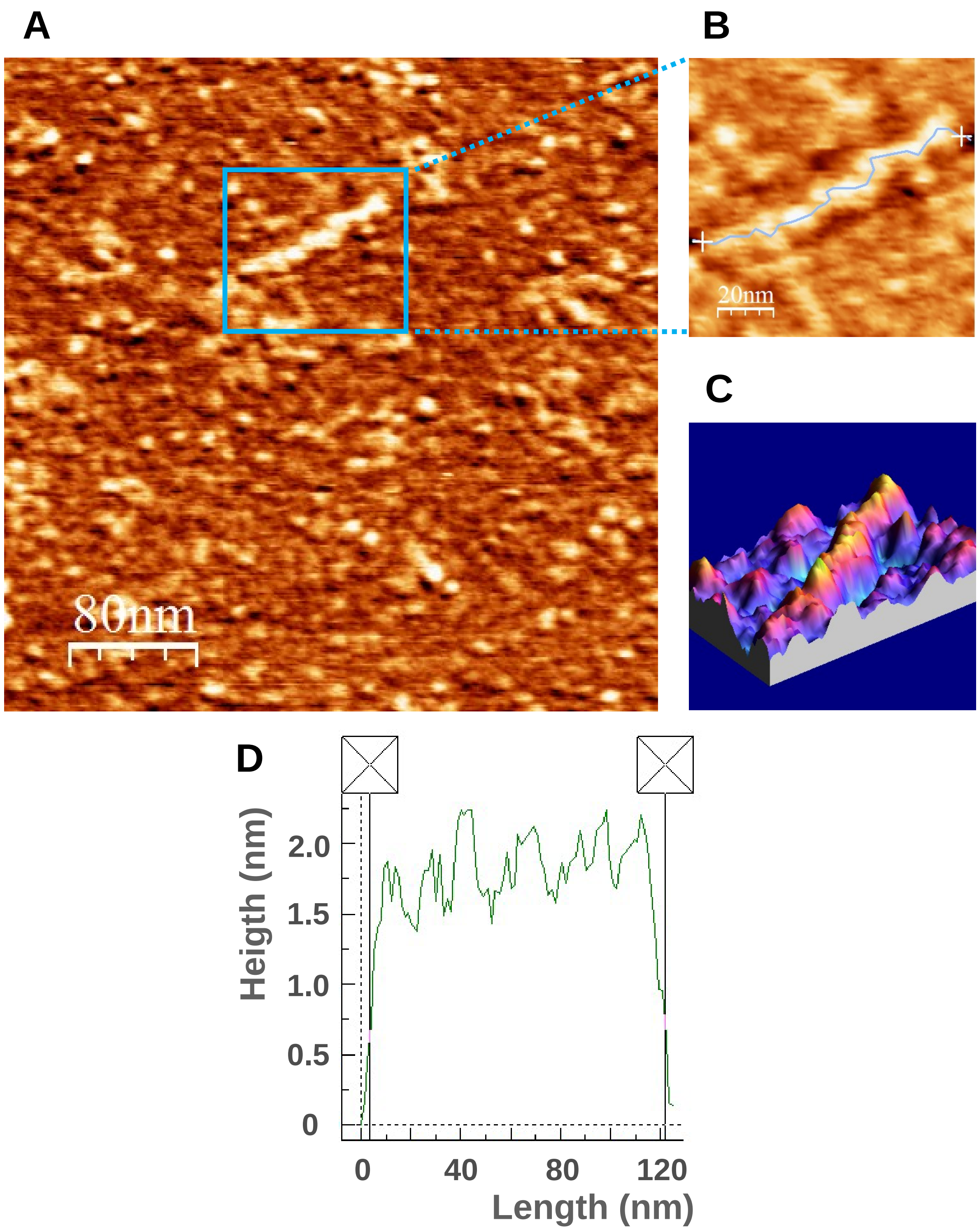

Supplementary Figure 2. Tapping mode AFM image of denatured IRES-574 RNA molecules in 80% formamide. (A) AFM image (400x400 nm) of denatured IRES-574 molecules deposited onto a pretreated mica-APTES surface, using an AFM tip with nominal curvature radius of 2 nm. **(B)** Zoom image (100x100 nm) of the molecule boxed in panel A, with a profile line along its length. **(C)** 3D representation of the selected molecule. **(D)** Length profile recorded following the line drawn in panel B, showing a contour distance of 118 nm (76 % of the theoretical molecular length).

Supplementary Figure 3

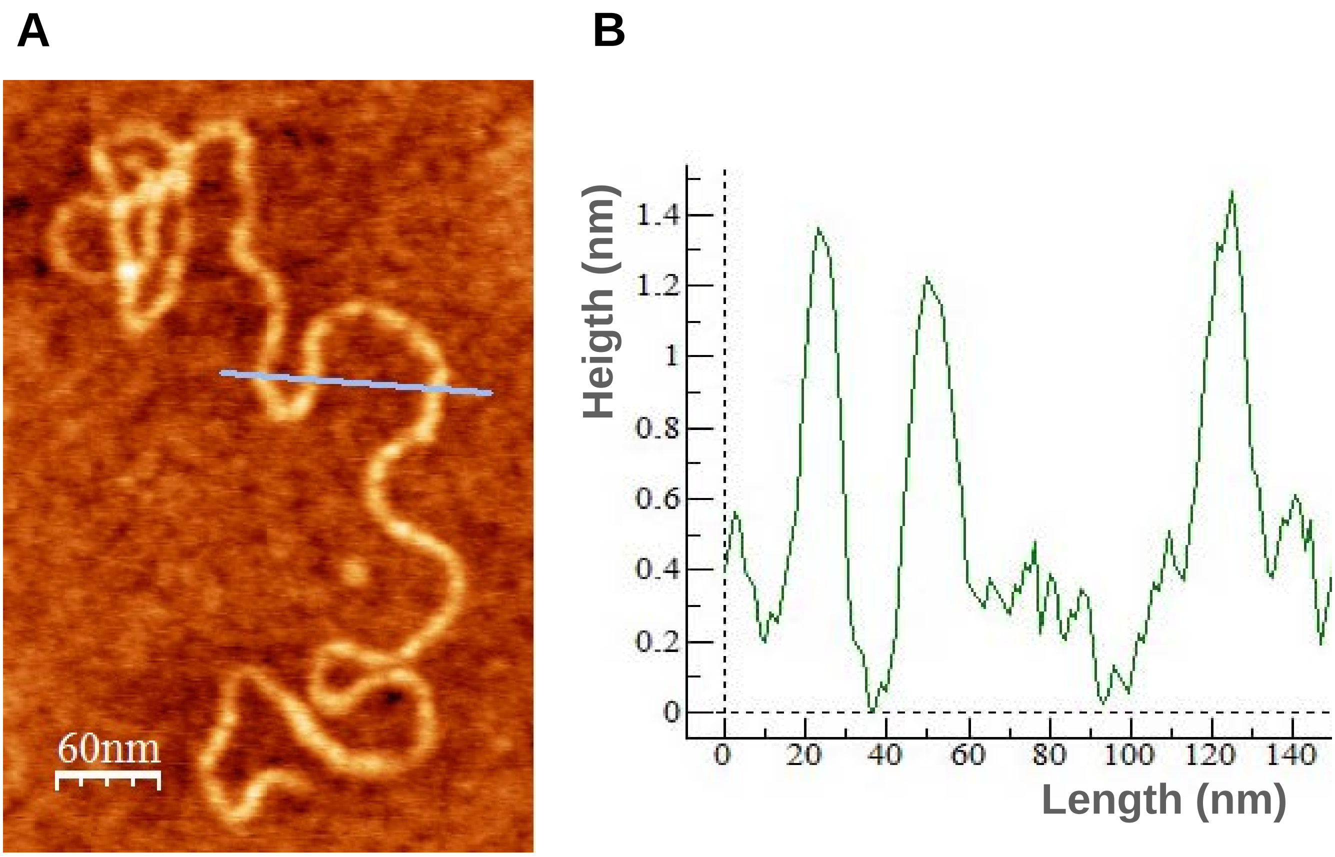

Supplementary Figure 3. AFM image of a control dsRNA molecule. (A) Tapping mode AFM image of the 4,579 bp-long genomic dsRNA of the totivirus L-A, using an AFM tip with nominal curvature radius of 2 nm. **(B)** Profile of the imaged molecule, showing a height of 1.2-1.4 nm, about 57% of the theoretical 2.3 nm diameter of the A-RNA double helix in solution. Such an apparent flattening of the molecule could be due to the pressure exerted by the AFM tip on the RNA sample. Other possible reasons why height values are lower than expected include the own roughness of the APTES films and the dry conditions of the imaging process.

Supplementary Figure 4

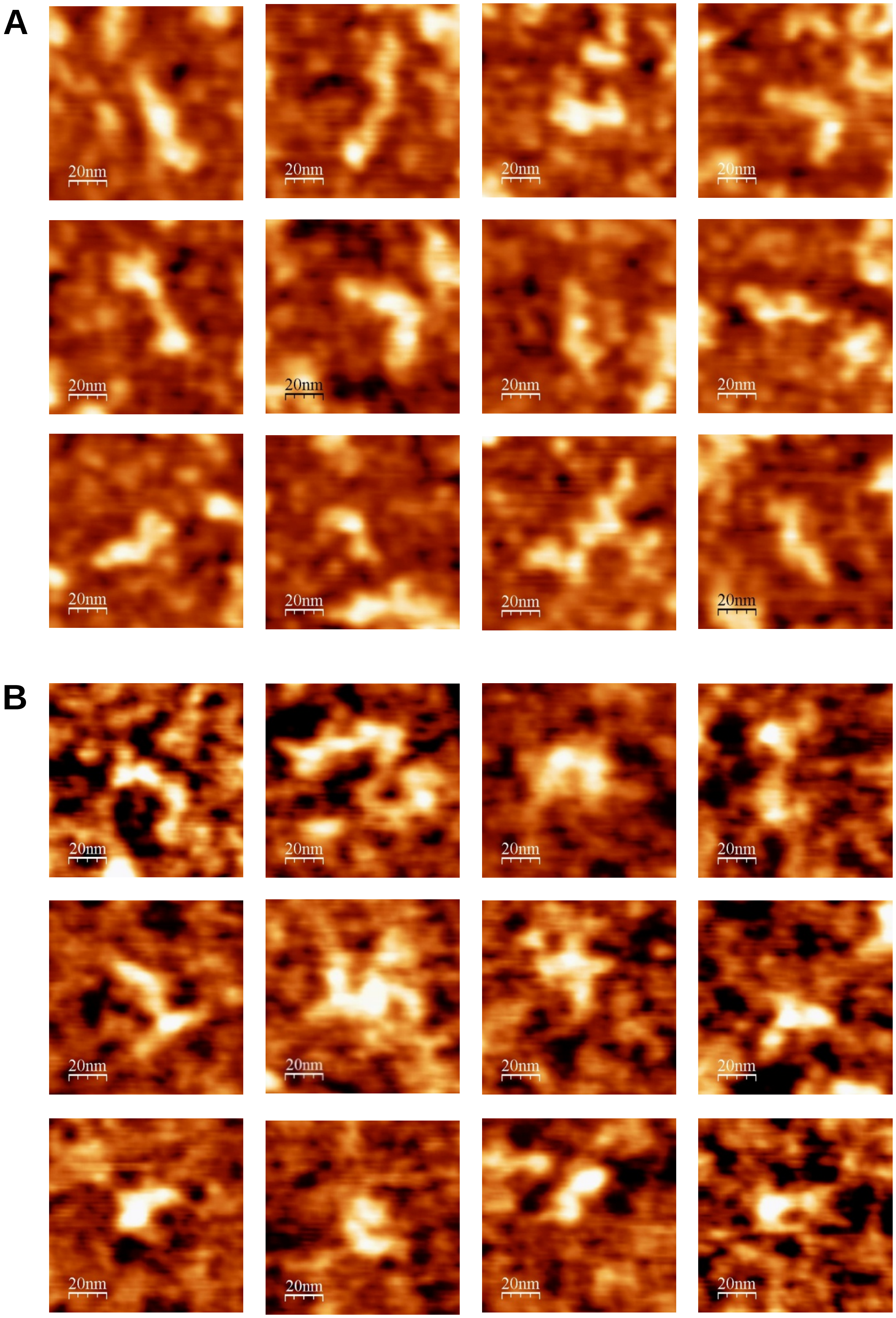

Supplementary Figure 4 (cont.)

C

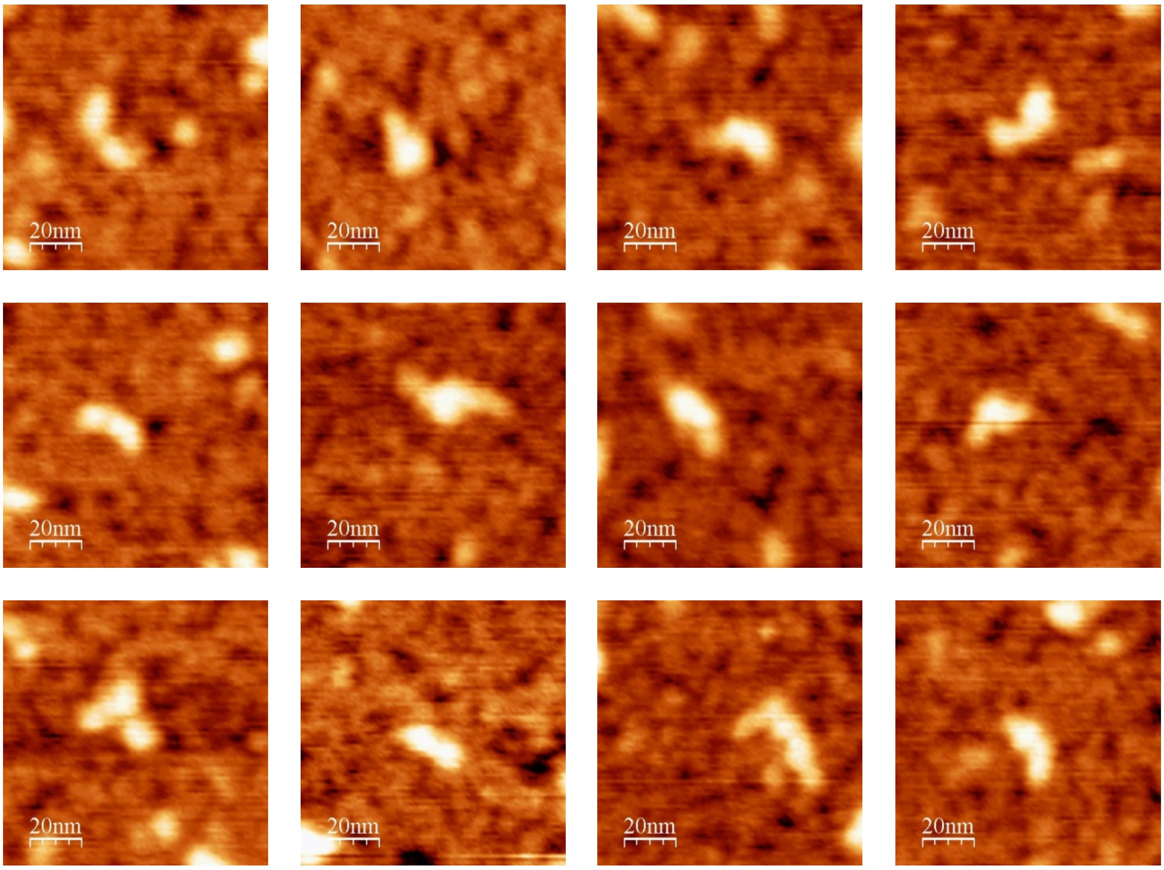

D

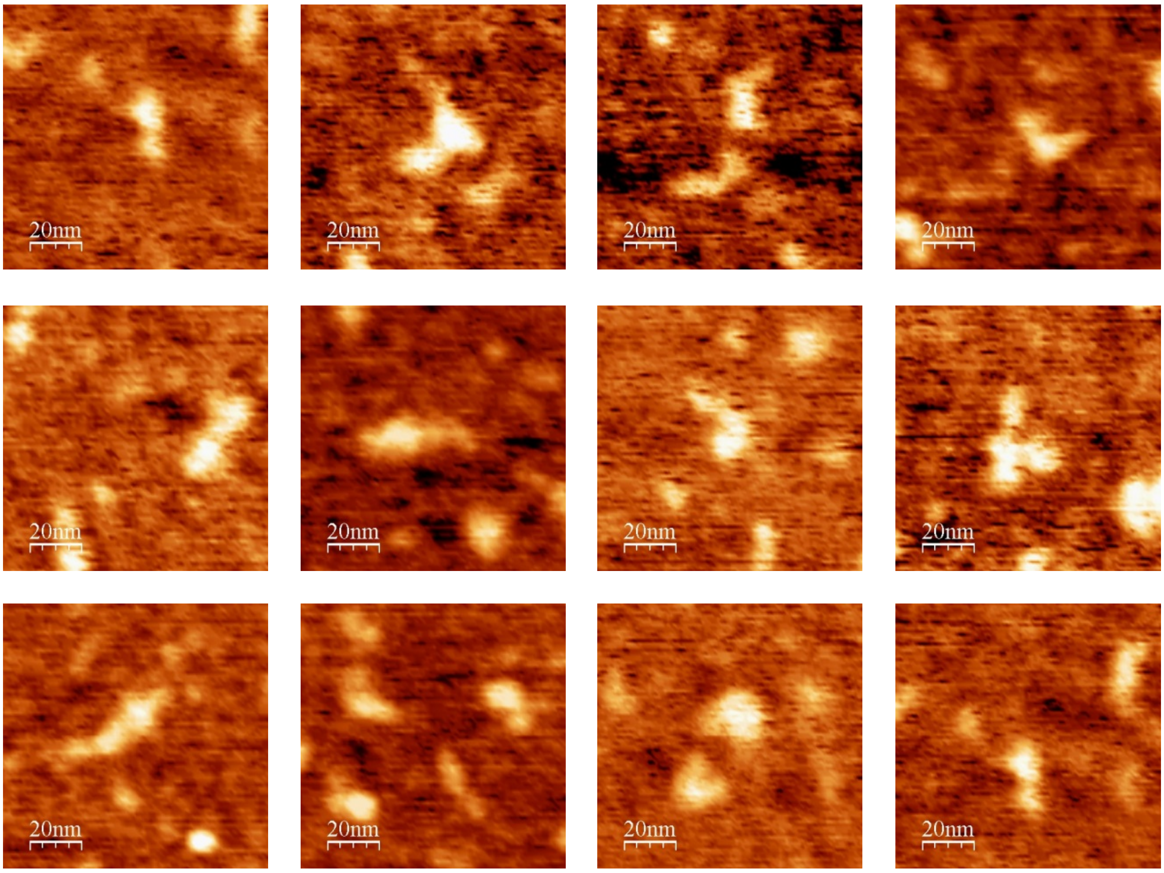

Supplementary Figure 4 (cont.)

E

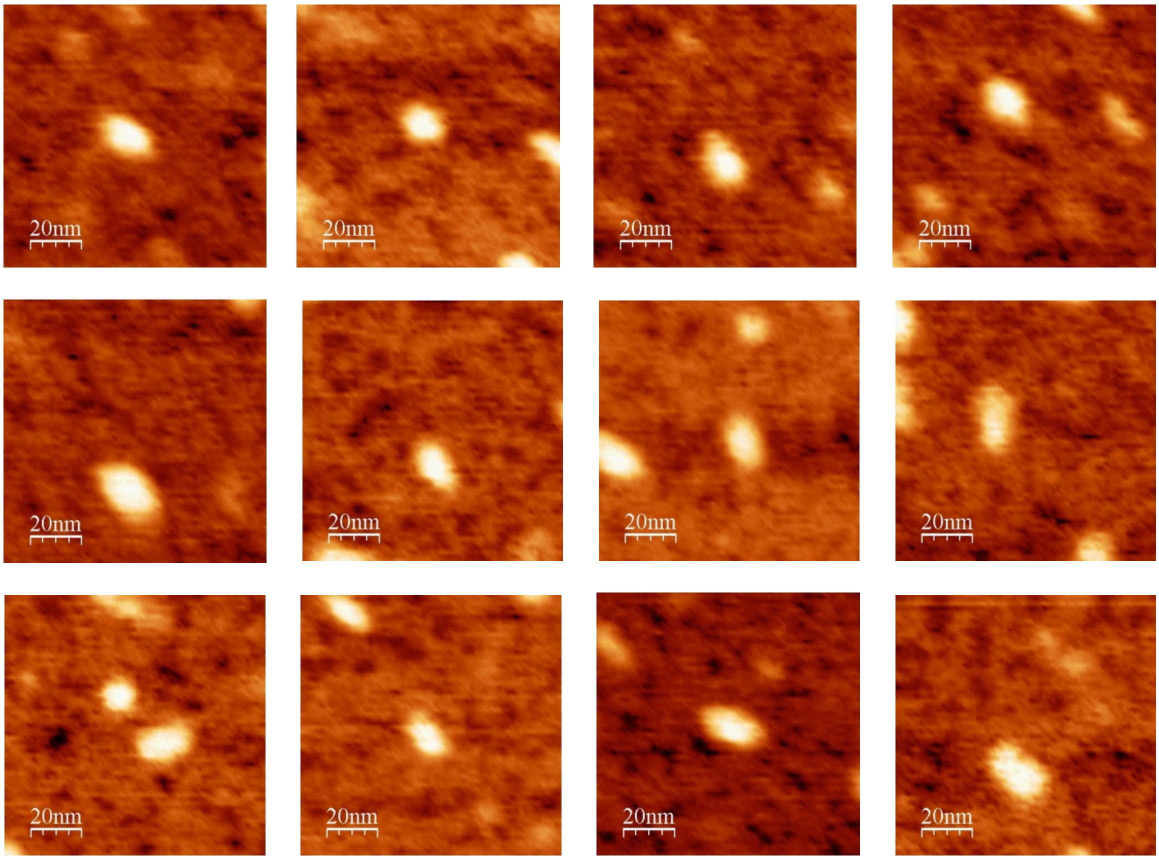

Supplementary Figure 4. Representative AFM images (100x100 nm) of individual IRES-574 molecules under different ionic conditions. (A) Molecules in folding buffer (100 mM NaCl and 100 mM HEPES pH 7.5) without added $MgCl_2$. Extended morphologies are observed, showing some bumps likely corresponding to elements of secondary structure. **(B)** Folding buffer supplemented with 2 mM Mg^{2+}. Extended shapes (upper row) coexist with molecules showing complex, branched morphologies where up to 4 arms protrude from a central axis (medium row), and angular molecules showing 2 arms (lower row). **(C)** Folding buffer supplemented with 4 mM Mg^{2+}. Compact, comma-shaped molecules are imaged. **(D)** Folding buffer supplemented with 6 mM Mg^{2+}. Morphologies analogous to those found at 4 mM Mg^{2+} are dominant, together with elliptical-shaped molecules. **(E)** Folding buffer supplemented with 10 mM Mg^{2+}. Very compact molecules with elliptical morphologies are imaged. The nominal curvature radius of the AFM tips was 2 nm. See main text for further details.

Supplementary Figure 5

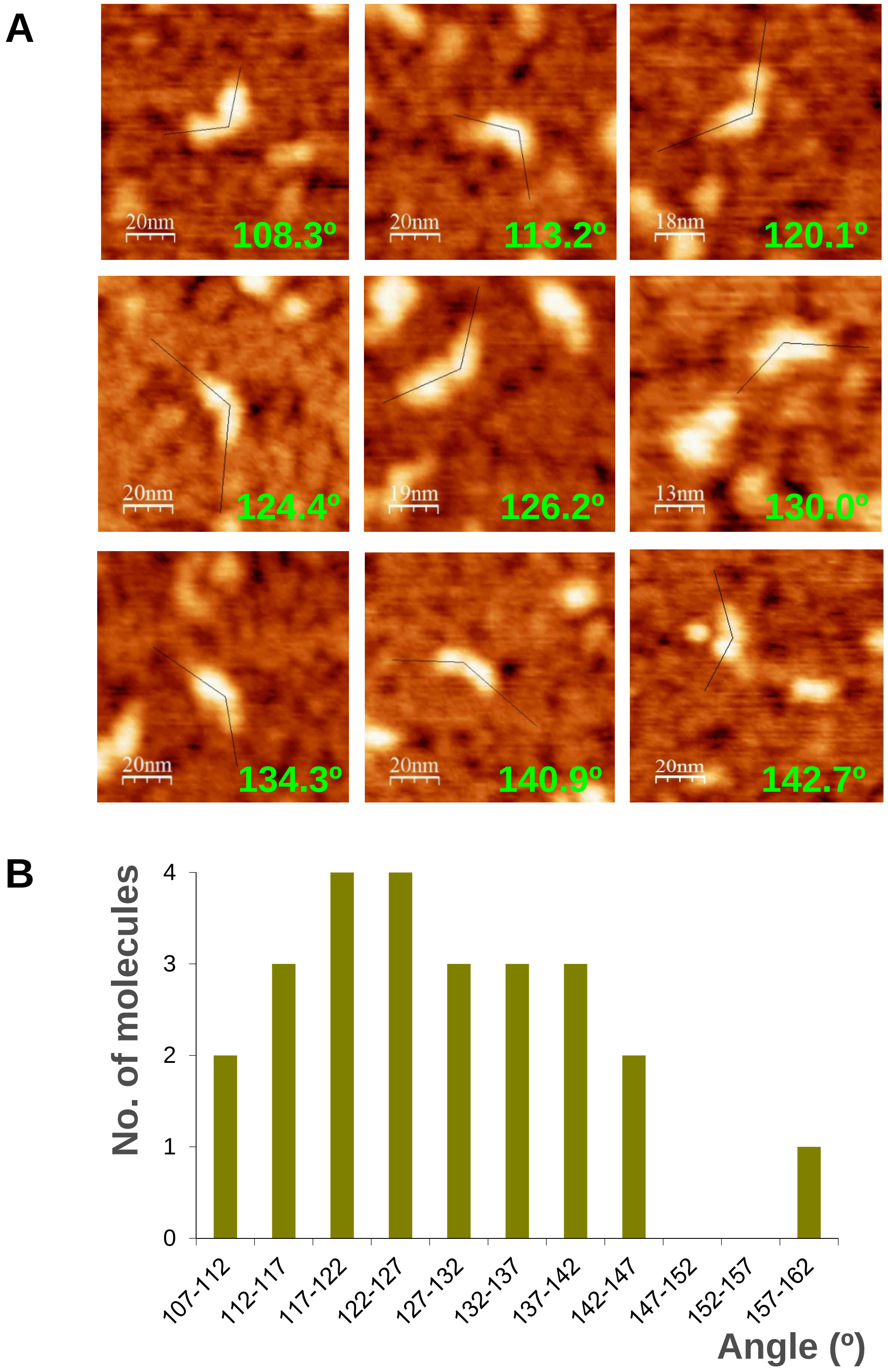

Supplementary Figure 5. Distribution of the angles formed between the two arms imaged in IRES-574 molecules at 4 mM Mg^{2+}. (A) Selection of nine representative images (100x100 nm), showing the angle between the two imaged arms. The nominal curvature radius of the AFM tips was 2 nm. **(B)** Graphical representation of the angular distribution, computed over 25 molecules that showed the correct orientation on the surface.

Supplementary Figure 6

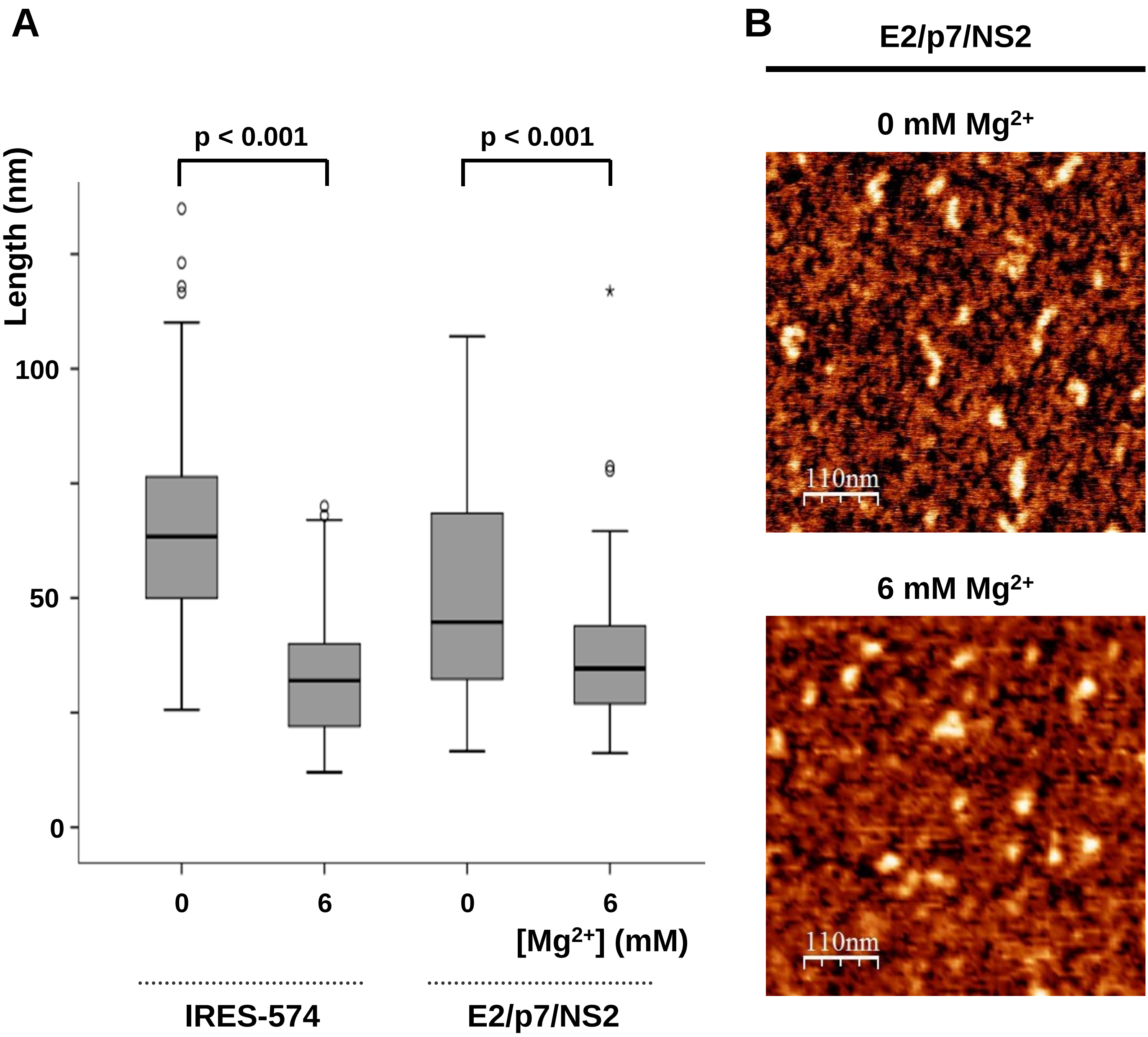

Supplementary Figure 6. Differential behavior of two HCV genomic regions in their Mg^{2+}-induced folding. (A) Box plot of the length distribution (computed over 100 molecules) of the transcripts IRES-574 and E2/p7/NS2 in a folding buffer either in the absence of Mg^{2+} or supplemented with 6 mM Mg^{2+}. Boxes represent 25-75 percentile range, vertical lines span 10-90 percentile range, and the horizontal bar within the box represent the median. Outlier values are depicted as circles (mild outliers) and asterisks (extreme outliers). Statistical p values are shown. Both sequences exhibited statistically significant reductions in their median length values when Mg^{2+} concentration was increased from 0 to 6 mM. However, this effect was much more pronounced in IRES-574 (49.2% reduction of the median values, from 63 to 32 nm) than in E2/p7/NS2 (24.4% reduction, from 45 to 34 nm). **(B)** Representative AFM images (550x550 nm) of E2/p7/NS2 molecules in folding buffer without Mg^{2+} (upper panel) and supplemented with 6 mM Mg^{2+} (lower panel). The nominal curvature radius of the AFM tips was 2 nm.

Supplementary Figure 7

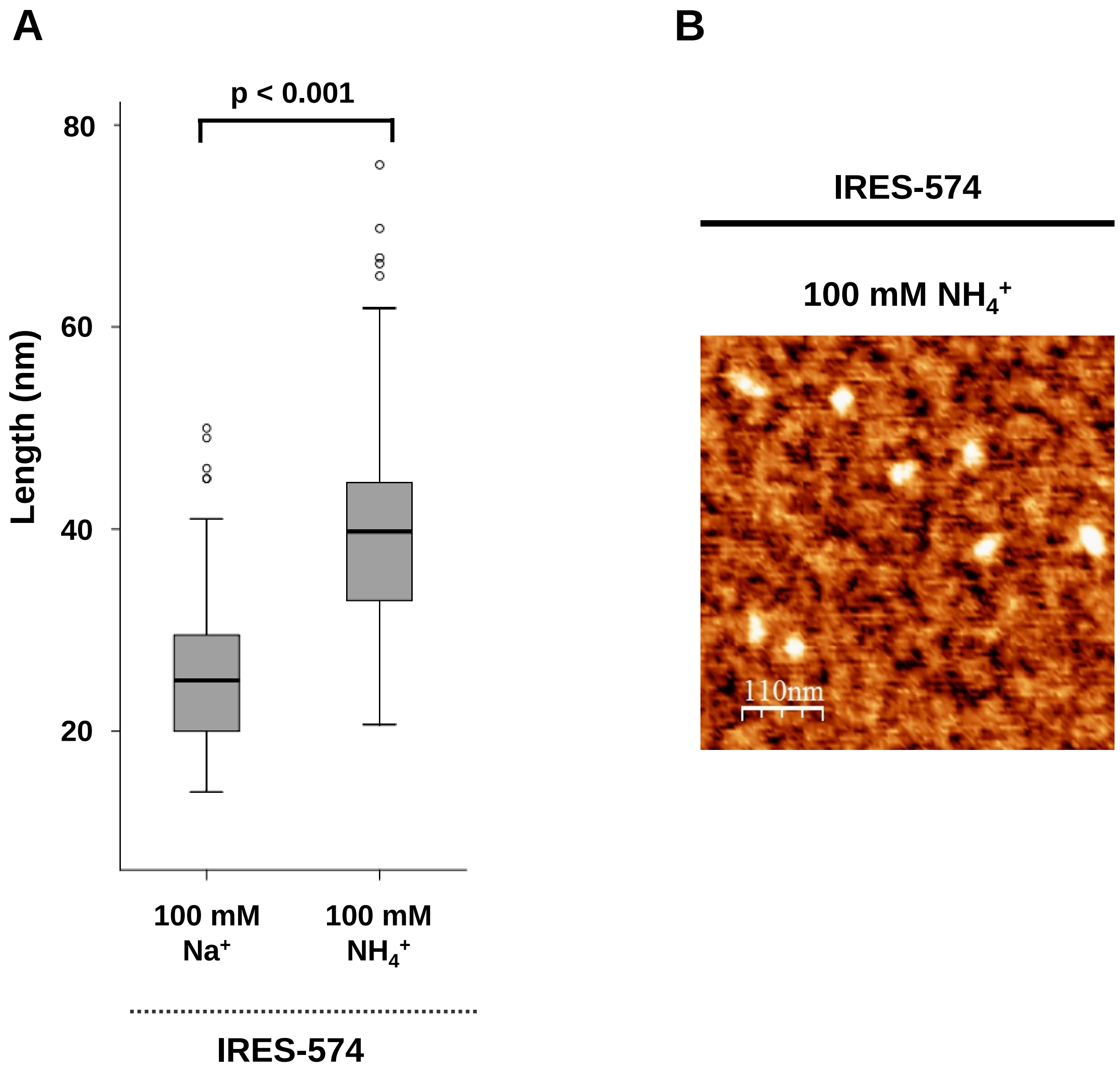

Supplementary Figure 7. Differential compaction of the IRES-574 molecule induced by two monovalent cations. (A) Box plot of the length distribution (computed over 100 molecules) of the transcript IRES-574 incubated in folding buffer containing 100 mM HEPES pH 7.5 and 10 mM $MgCl_2$, supplemented with either 100 mM NaCl or 100 mM NH_4Cl. Boxes represent 25-75 percentile range, vertical lines span 10-90 percentile range, and horizontal bar within the box represent the median. Mild outlier values are depicted as circles, and statistical p values are shown. The lower compactness of the molecules incubated in NH_4^+-containing buffer with respect to those folded under Na^+ was evidenced by their median lengths (39.5 nm and 25 nm, respectively). **(B)** Representative AFM image (550x550 nm) of IRES-574 molecule in folding buffer containing 100 mM NH_4Cl. The nominal curvature radius of the AFM tip was 2 nm.

Supplementary Figure 8

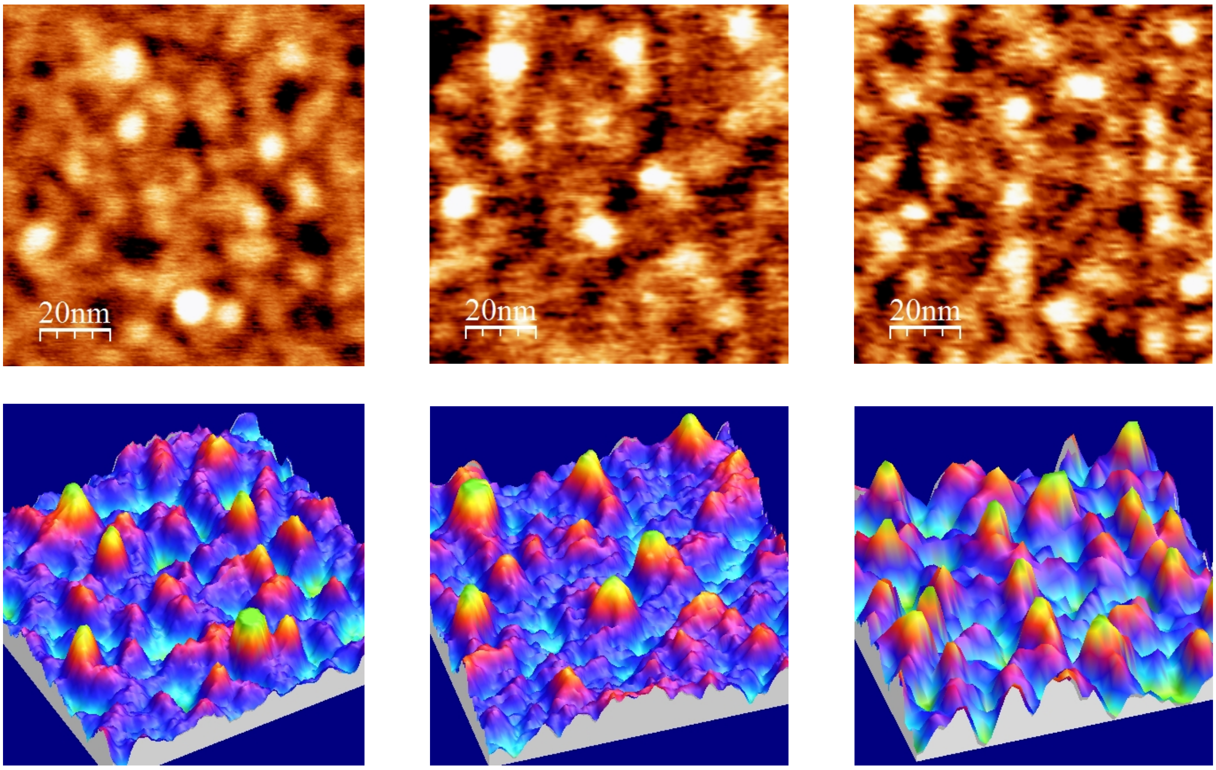

Supplementary Figure 8. AFM images of miR-122 molecules. MicroRNA miR-122 molecules at 15 nM concentration were incubated in a buffer composed of 100 mM NaCl, 100 mM HEPES pH 7.5, and 6 mM $MgCl_2$. Compact and elliptical shapes were imaged, with very homogeneous lengths of 10-14 nm. Three different AFM fields (100x100 nm) are shown, together with their corresponding 3D representation. The nominal curvature radius of the AFM tips was 2 nm.

Supplementary Figure 9

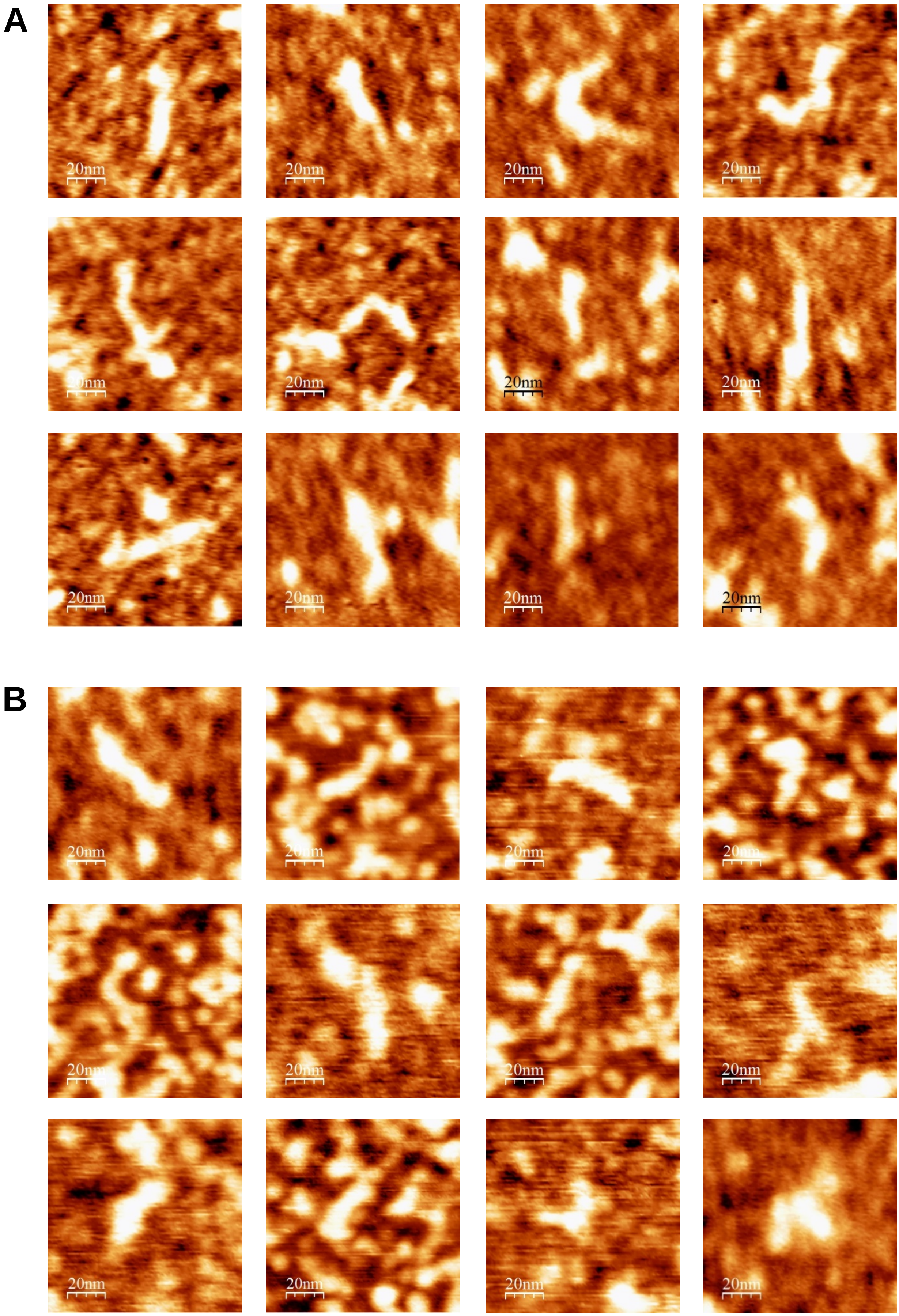

Supplementary Figure 9 (cont.)

C

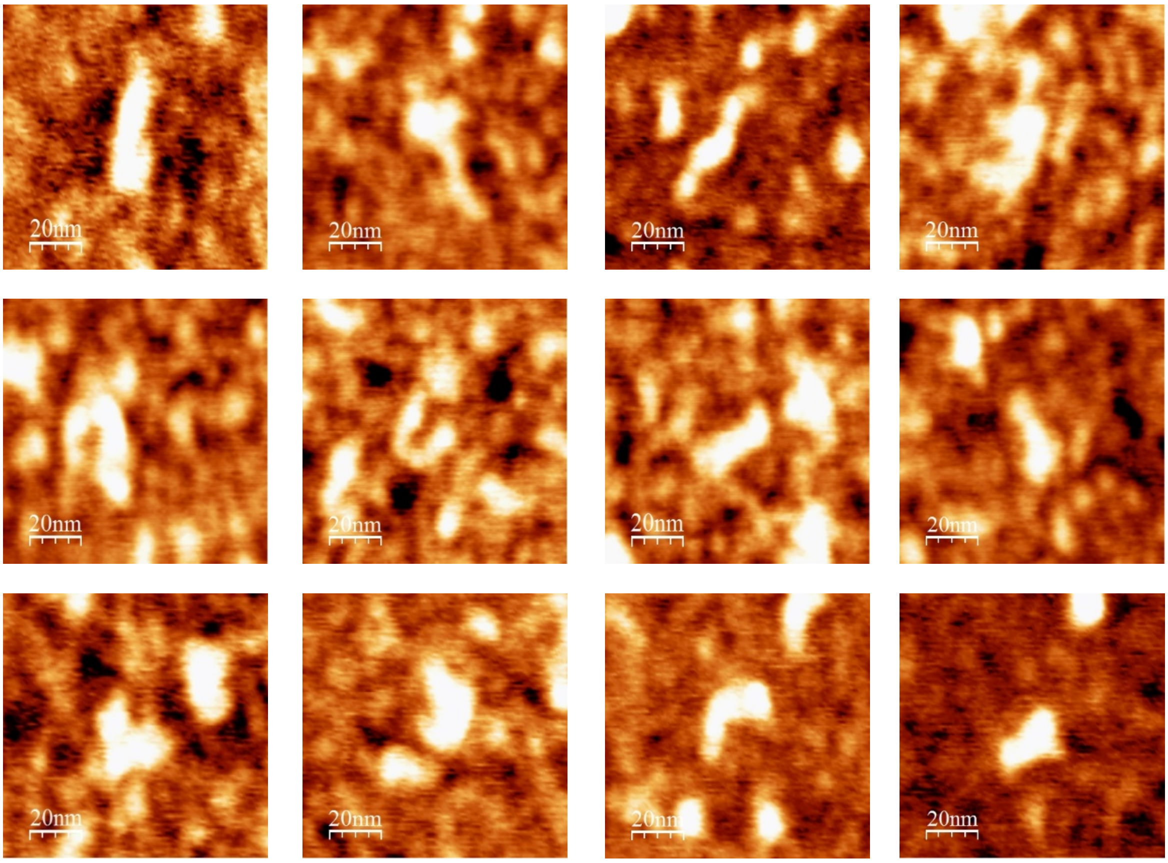

D

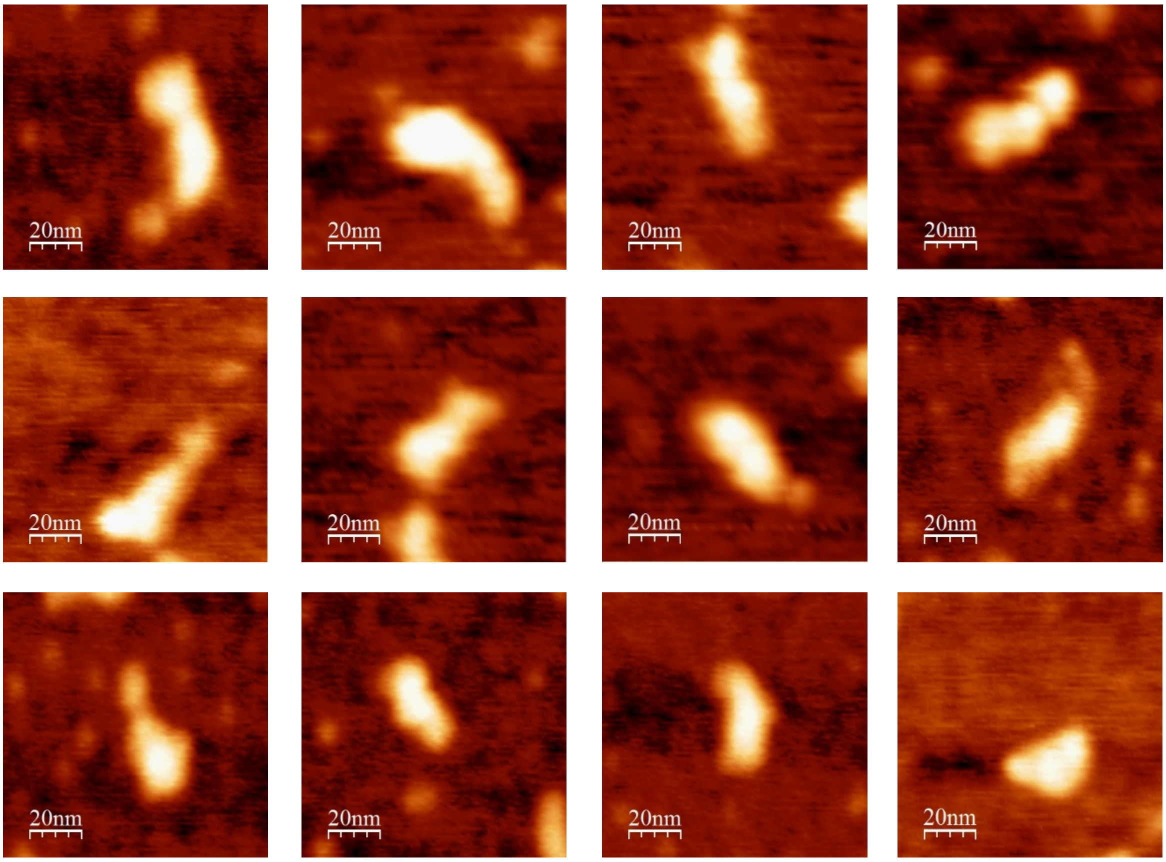

Supplementary Figure 9 (cont.)

E

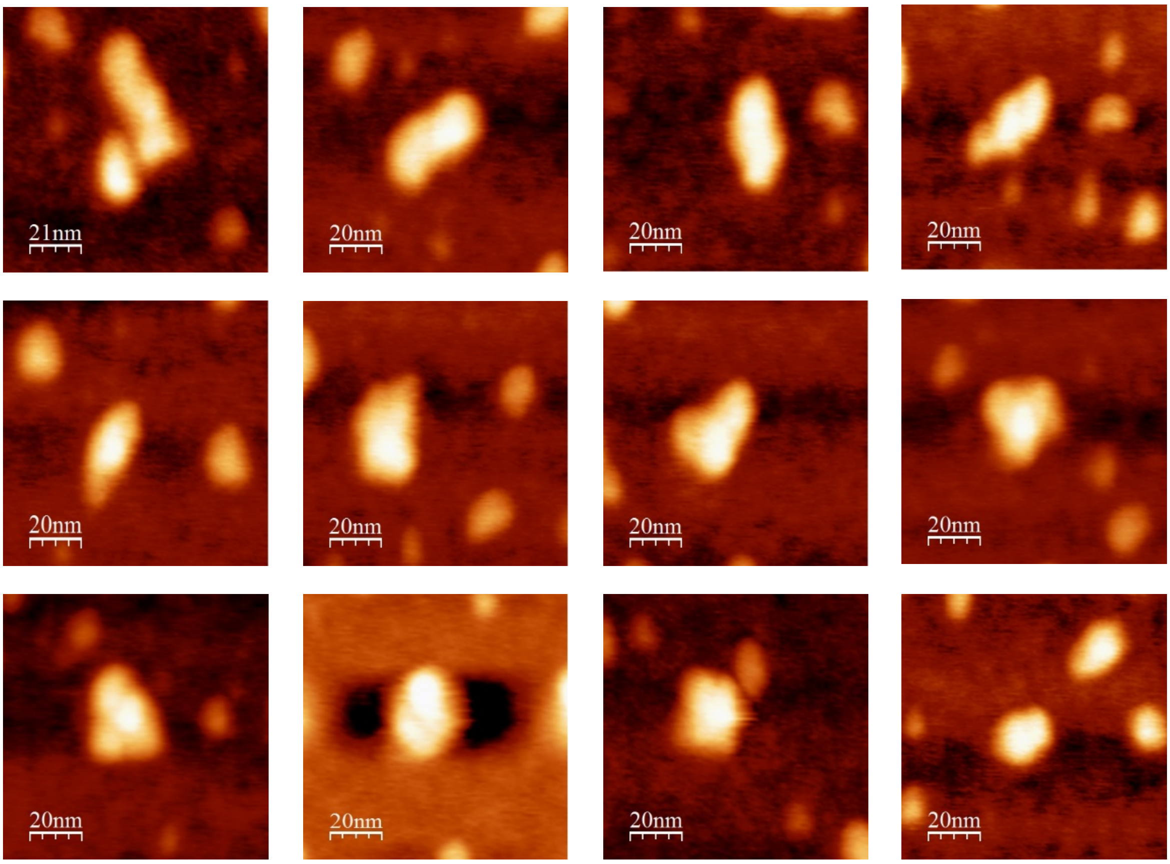

Supplementary Figure 9. Representative AFM images (100x100 nm) of individual IRES-574/miR-122 complexes under different ionic conditions. (A) Molecules in folding buffer (100 mM NaCl and 100 mM HEPES pH 7.5) without added $MgCl_2$. Extended and angular morphologies are observed. **(B)** Folding buffer supplemented with 2 mM Mg^{2+}. Elongated and zigzagging molecules are imaged. **(C)** Folding buffer supplemented with 4 mM Mg^{2+}. Extended and angular shapes (upper-left panels) coexist with molecules showing branched morphologies, as well as a fraction of triangular and comma-shaped ones (lower-right panels). **(D)** Folding buffer supplemented with 6 mM Mg^{2+}. Partially elongated and angular molecules are imaged, together with comma-shaped and more compact molecules. **(E)** Folding buffer supplemented with 10 mM Mg^{2+}. Some short, linear molecules are imaged, although most of the AFM fields are dominated by compact morphologies with triangular and elliptical shapes. The nominal curvature radius of the AFM tips was 2 nm. See main text for further details.
